# Supplementary material for: Rapamycin rescues mitochondrial myopathy via coordinated activation of autophagy and lysosomal biogenesis
Source: EMBO Mol Med. 2018 Oct 11;10(11):e8799. doi: 10.15252/emmm.201708799 (PMC6220341; doi:10.15252/emmm.201708799)
Supplement: Supplementary file 1 — Appendix [file EMMM-10-e8799-s001.pdf]

Appendix

Appendix Figure S1

Appendix Figure S2

Appendix Figure S3

Appendix Figure S4

Appendix Figure S5

Appendix Figure S6

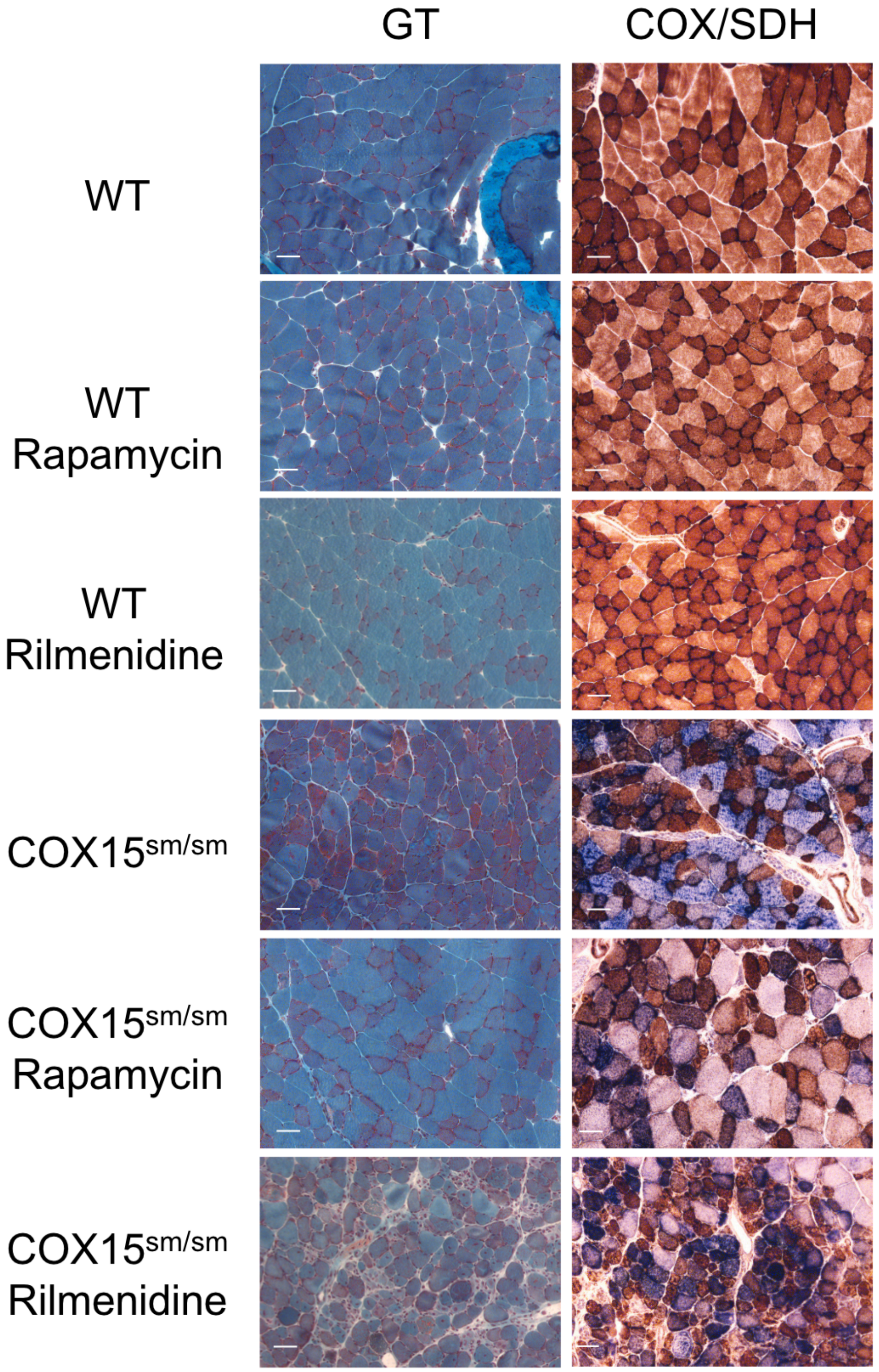

Appendix Figure S1. Histological and histochemical characterization of skeletal muscle in rapamycin-treated and untreated *Cox15$^{sm/sm}$* and *WT* mice.
GT: Gomori trichrome. **COX/SDH**: double histochemical staining for COX and SDH. White bars correspond to 50μm.

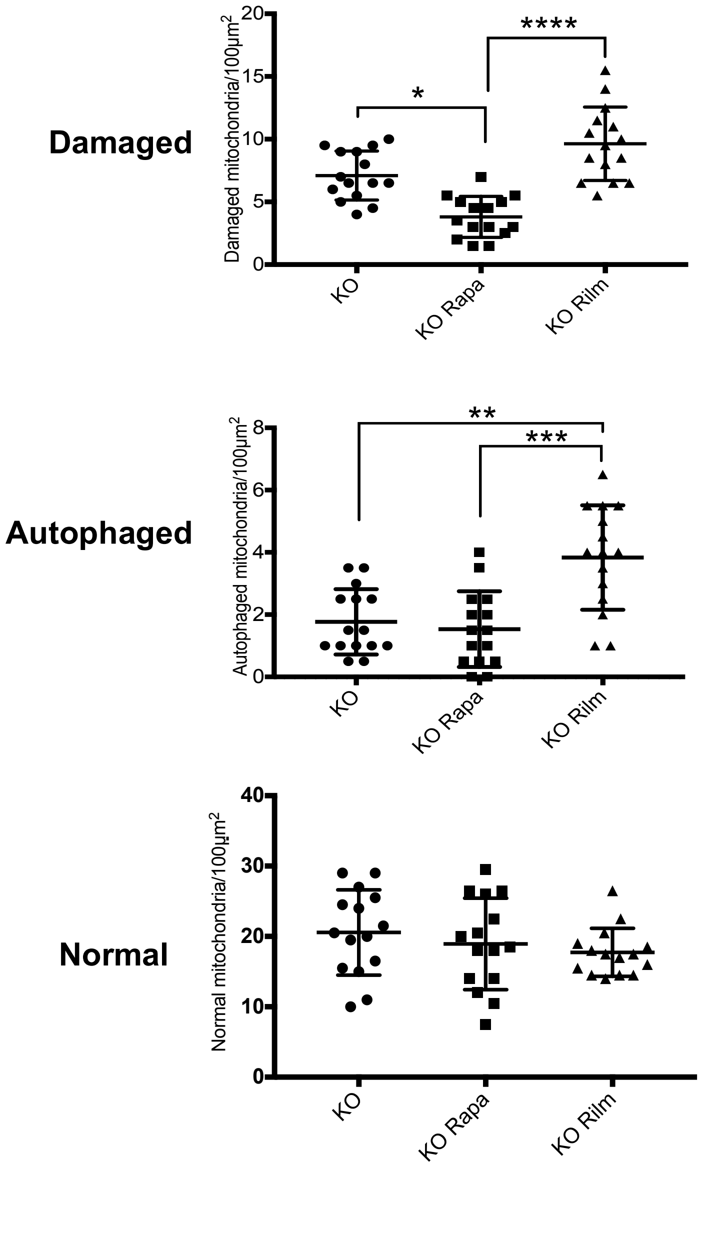

Appendix Figure S2. Quantification of damaged, autophaged and normal mitochondria in muscle samples.

Ten independent, low magnification fields for a total area of 855μm^2 were analyzed in 2 animals/group. Error bars represent SEM. The asterisks represent the significance levels calculated by two-way ANOVA with Tukey post-hoc multiple comparison test: Damaged mitochondria: *p=0.0123 (KO vs KO+rapa), ****p<0.0001 (KO+rapa vs KO+rilmenidine); Autophaged mitochondria: **p=0.0012 (KO vs KO+rilmenidine), ***=0.0003 (KO vs KO+rapa).

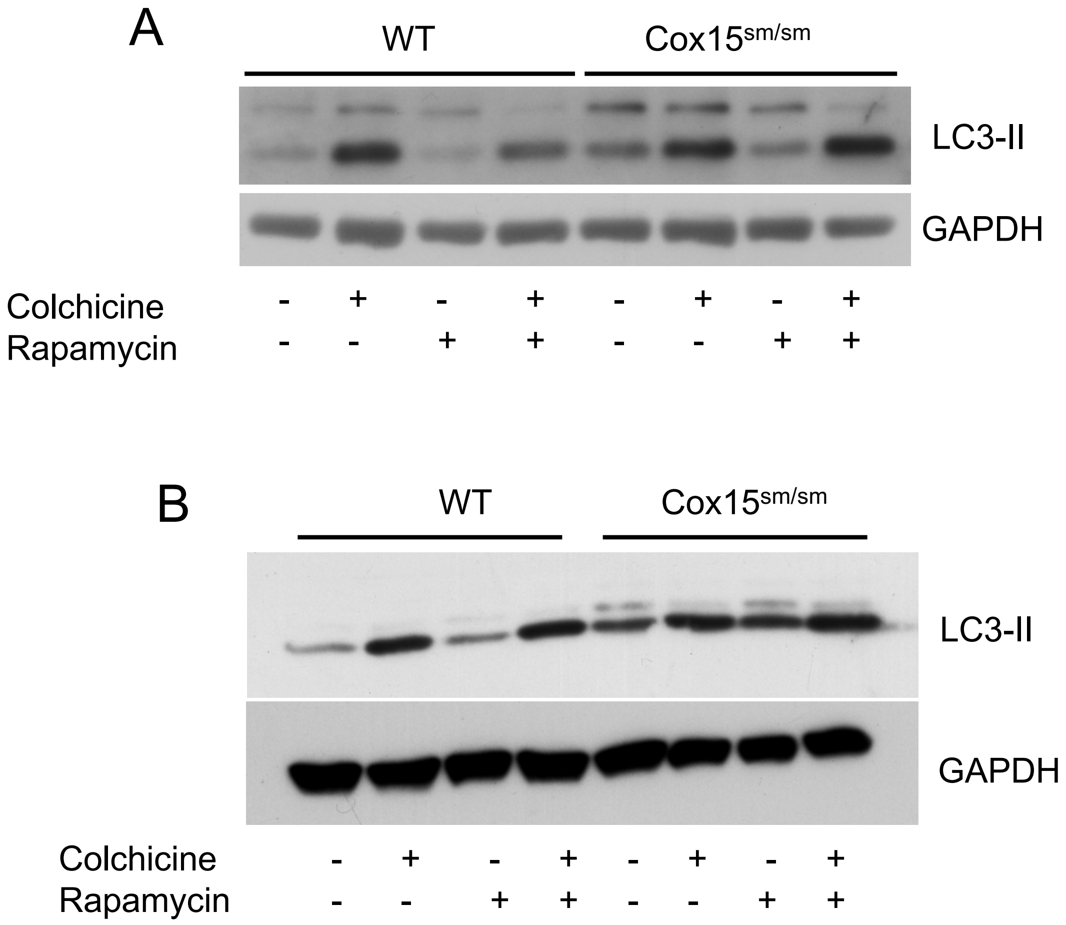

Appendix Figure S3. Additional WB analysis of autophagic flux.
Two additional western blot of *Cox15*$^{sm/sm}$ and WT muscles treated with colchicine, rapamycin, or rapamycin plus colchicine. Note that LC3-II was increased in *Cox15*$^{sm/sm}$ vs. *WT* samples. Colchicine did not increase LC3-II levels suggesting a block in the autophagic flux; rapamycin plus colchicine treated *Cox15*$^{sm/sm}$ mice showed higher levels of LC3-II, suggesting that rapamycin increased the autophagic flux in *Cox15*$^{sm/sm}$ muscles. Quantitative analysis is reported in figure 4C.

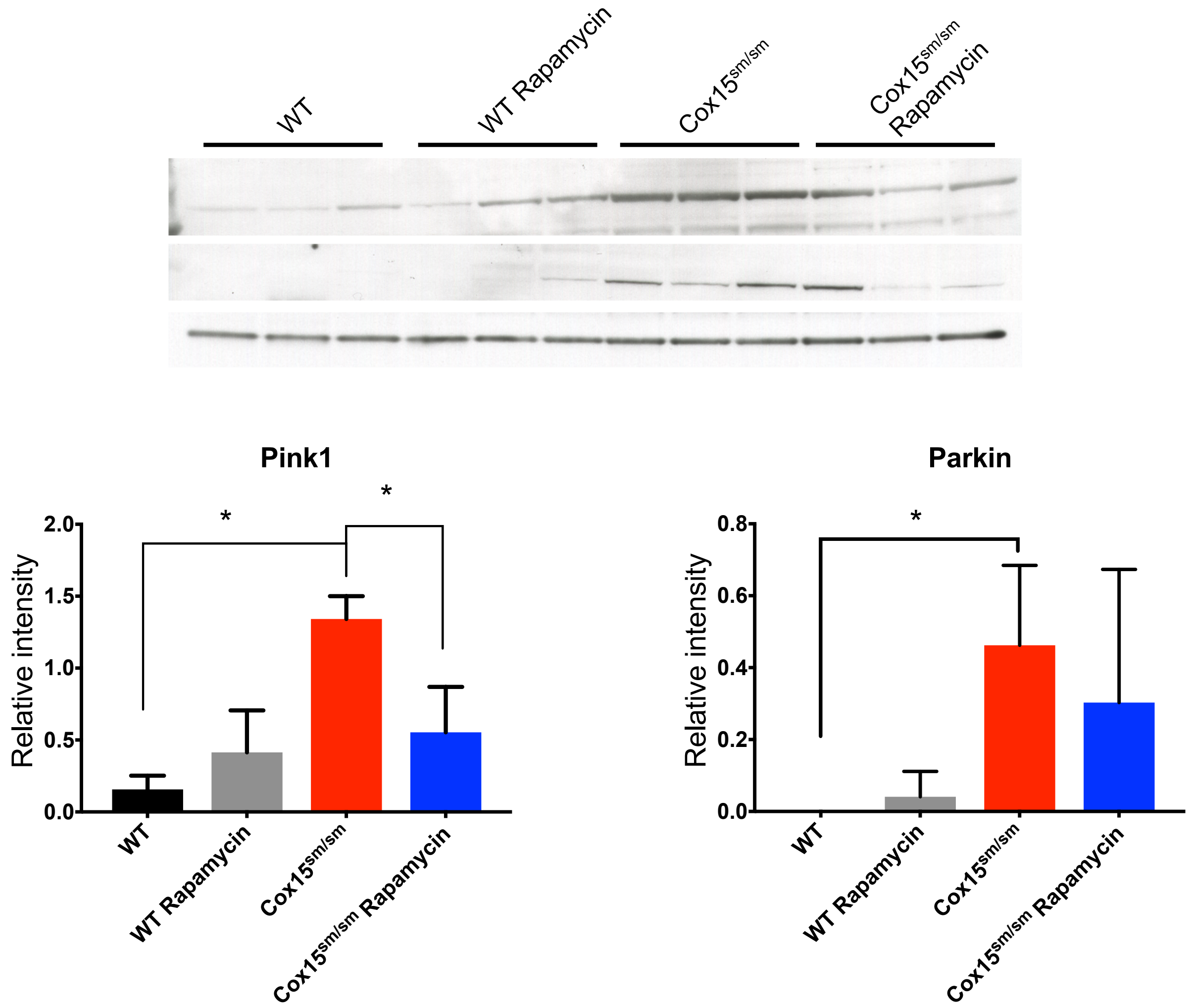

Appendix Figure S4. WB analysis of PINK1 and Parkin.
Black: untreated *WT*; grey: rapamycin-treated *WT*; red: untreated *Cox15*$^{sm/sm}$; blue: rapamycin-treated *Cox15*$^{sm/sm}$. Error bars represent SEM. The asterisks represent the significance levels calculated by one-way ANOVA with Tukey post-hoc multiple comparison test: *p=0,0141 (KO vs KO+rapa); **p=0.00212 (WT vs KO); *p=0.010 (WT vs KO), *p=0.05 (KO+rapa vs. KO). N=3/group.

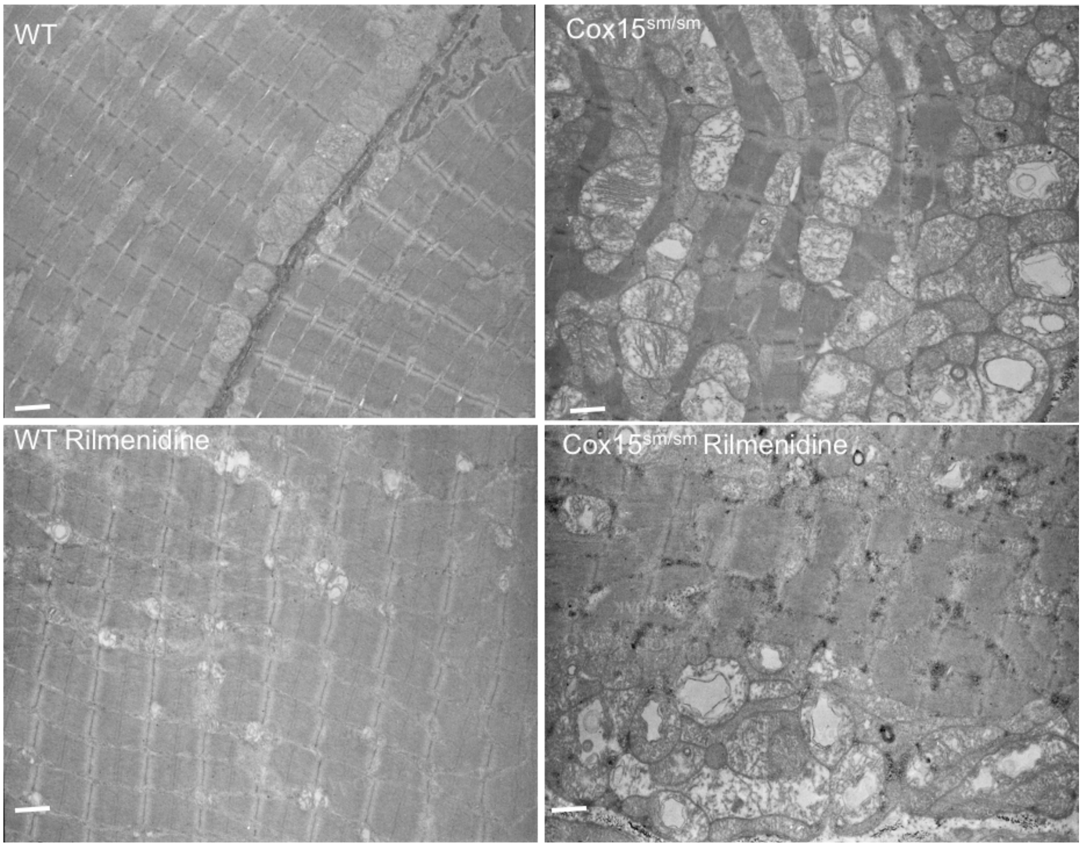

Appendix Figure S5. Rilmenidine does not improve mitochondrial ultrastructure in *Cox15^{sm/sm}* mice.

Altered mitochondria in *Cox15*$^{sm/sm}$ muscles persists in rilmenidine-treated *Cox15*$^{sm/sm}$ animals. Notably, several disrupted mitochondria can be detected also in rilmenidine-treated *WT* samples. The bar corresponds to 1136 nm (4400X).

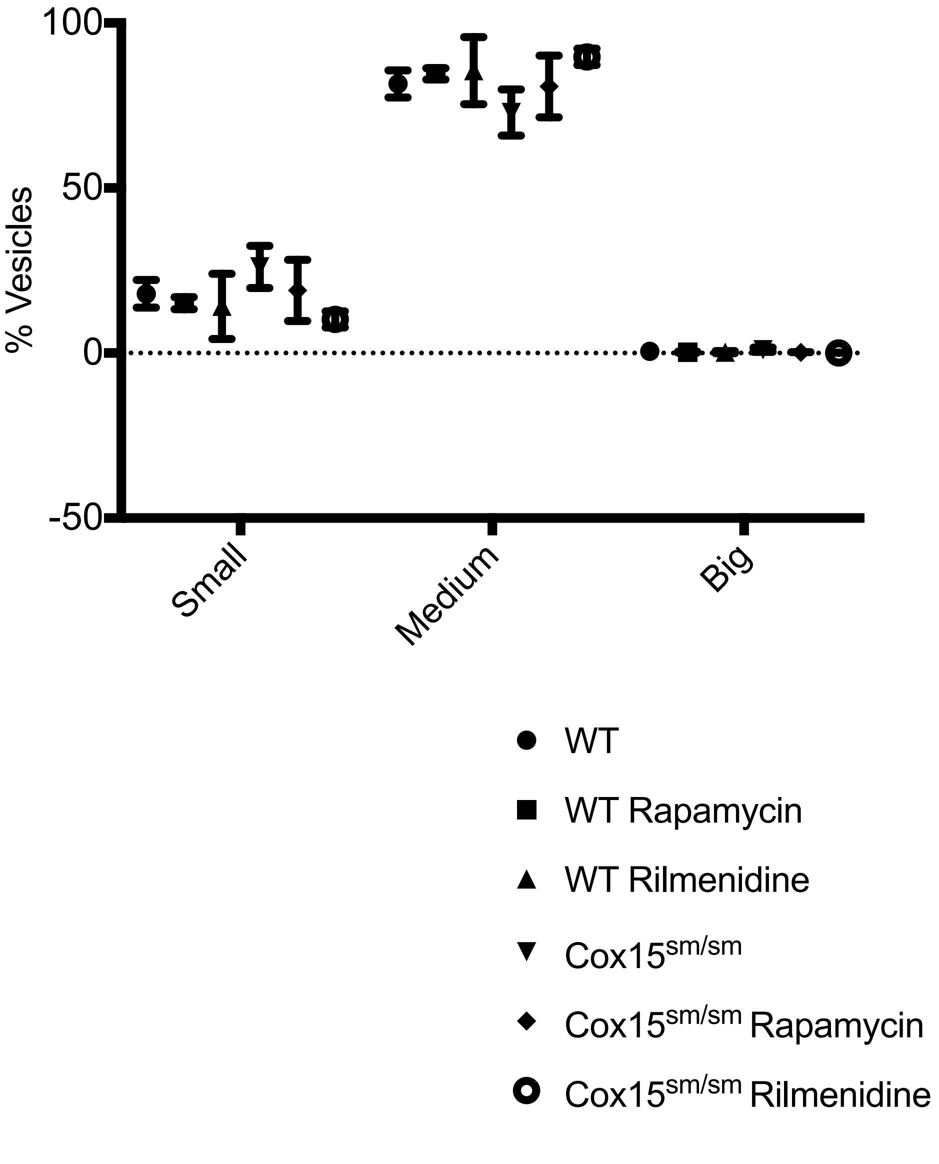

Appendix Figure S6. Quantification analysis of the size of LAMP1-positive vesicles. LAMP1 positive vesicles were classified as small if their diameter was <0.5μm, medium if the diameter was between >0.5<1.0μm, big if the diameter was >1.0μm
