# Supplementary figures and images for: Rapamycin rescues mitochondrial myopathy via coordinated activation of autophagy and lysosomal biogenesis
Source: EMBO Mol Med. 2018 Oct 11;10(11):e8799. doi: 10.15252/emmm.201708799 (PMC6220341; doi:10.15252/emmm.201708799)

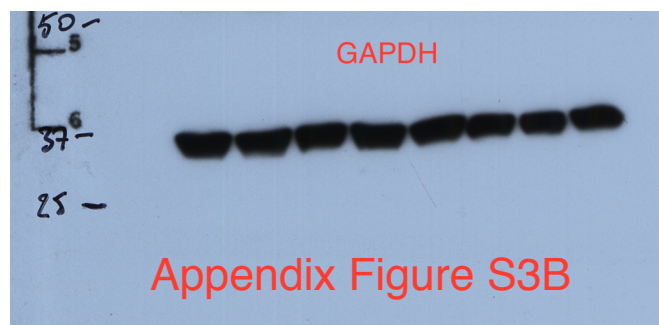

Supplement: Supplementary file 2 — Source Data for Appendix [file EMMM-10-e8799-s006.zip › emmm201708799-sup-0006-SDataFigS3.pdf]

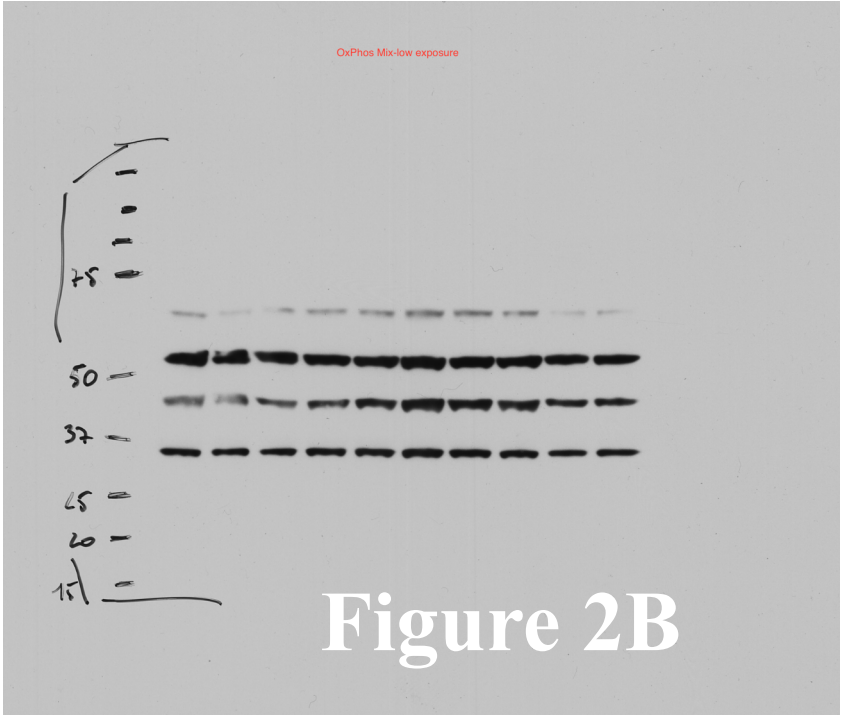

OxPhos Mix-High Exposure-CoxIV

## Figure 2B

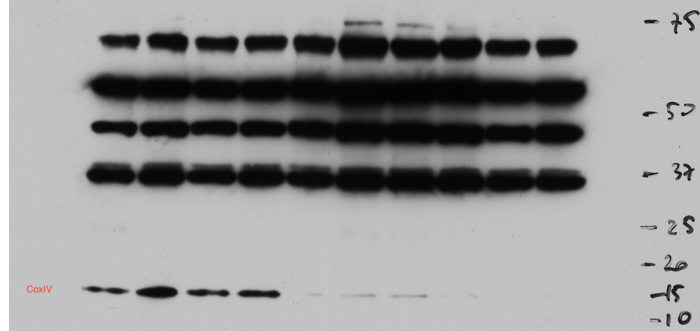

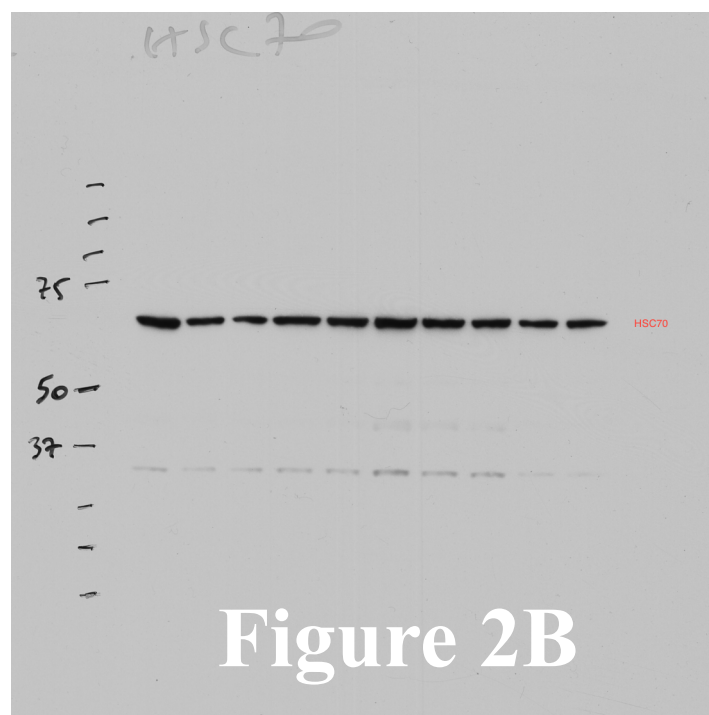

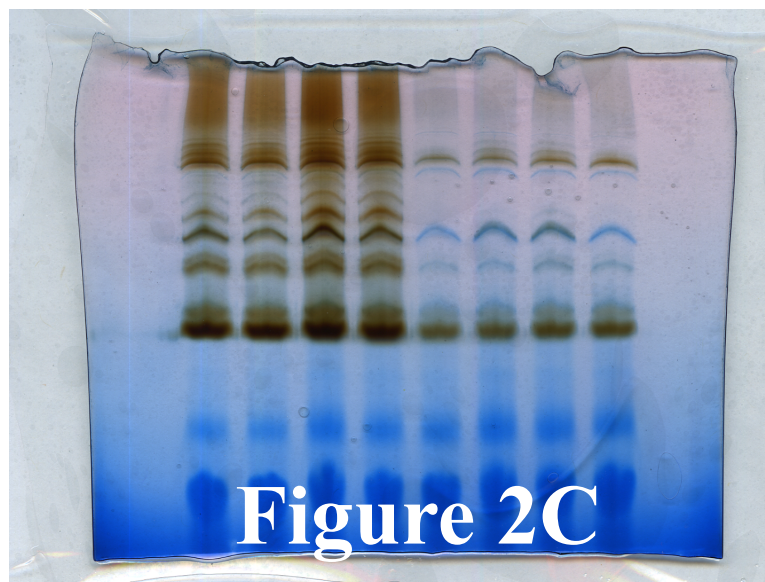

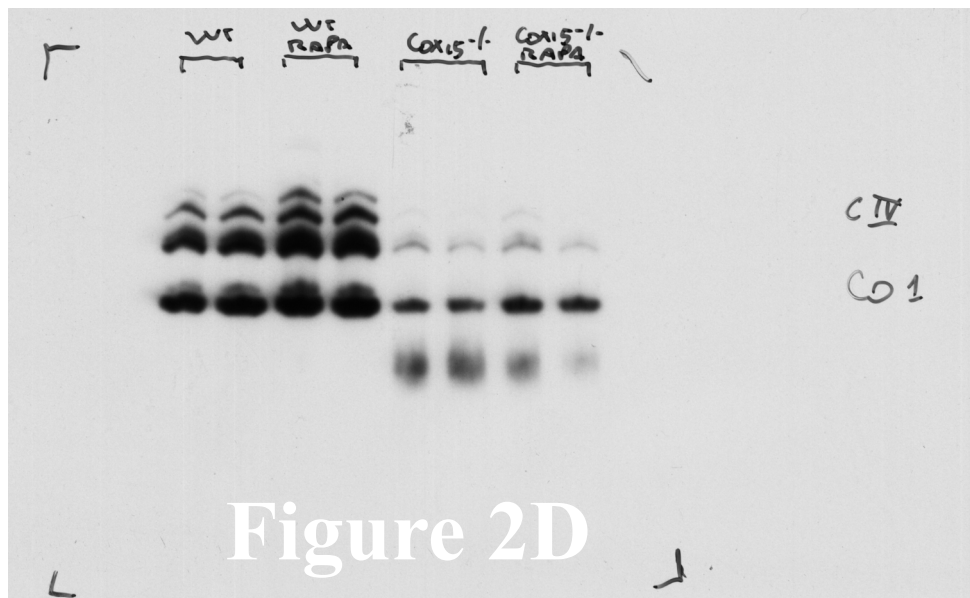

Supplement: Supplementary file 5 — Source Data for Figure 2 [file EMMM-10-e8799-s003.pdf]

# S6 and P-S6 Figure 4A

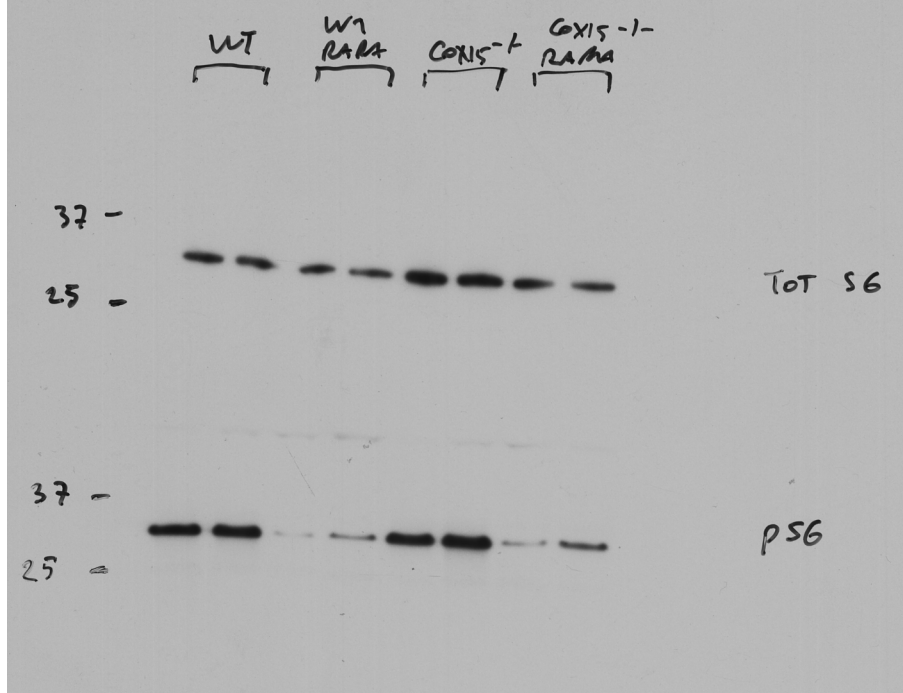

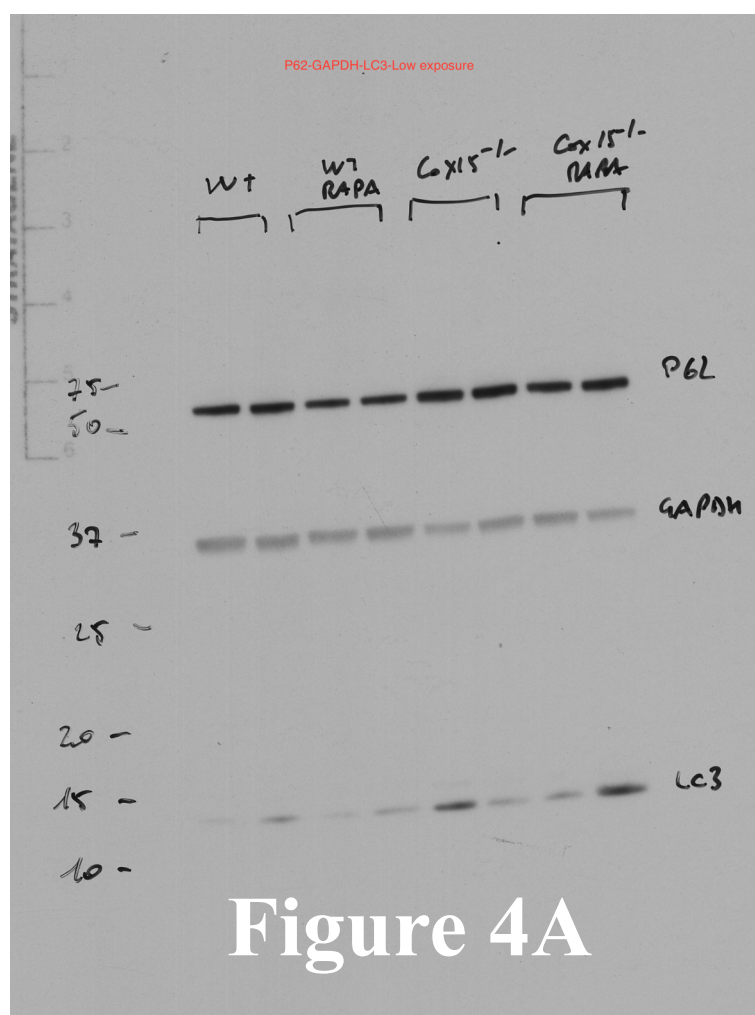

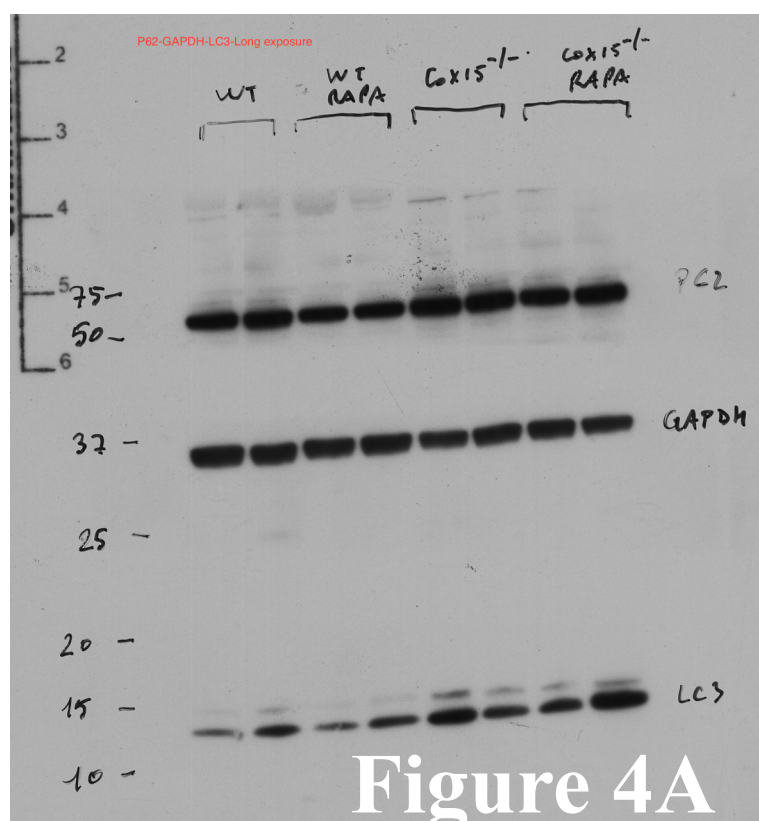

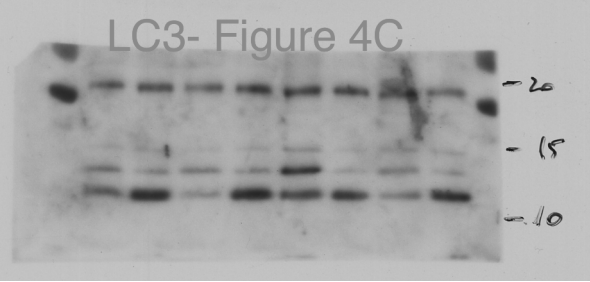

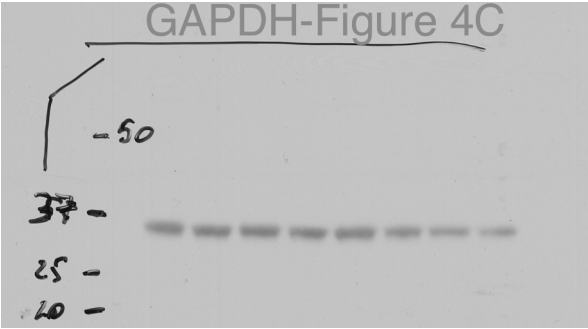

Supplement: Supplementary file 6 — Source Data for Figure 4 [file EMMM-10-e8799-s004.pdf]
