# Supplementary material for: Rapamycin rescues mitochondrial myopathy via coordinated activation of autophagy and lysosomal biogenesis
Source: EMBO Mol Med. 2018 Oct 11;10(11):e8799. doi: 10.15252/emmm.201708799 (PMC6220341; doi:10.15252/emmm.201708799)
Supplement: Supplementary file 4 — Source Data for Figure 1 [file EMMM-10-e8799-s002.pdf]

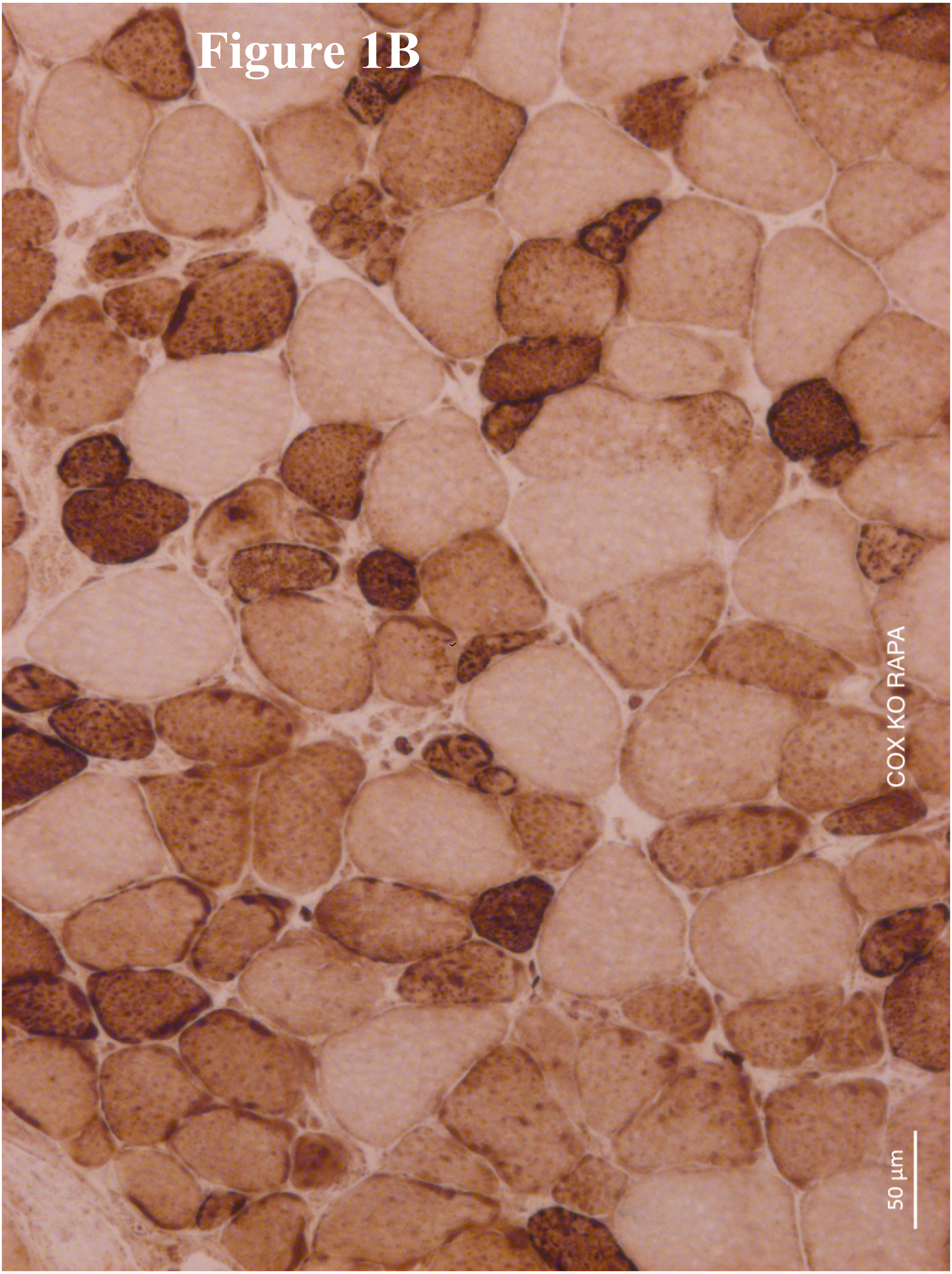
Figure 1B
COX KO RAPA
50 µm

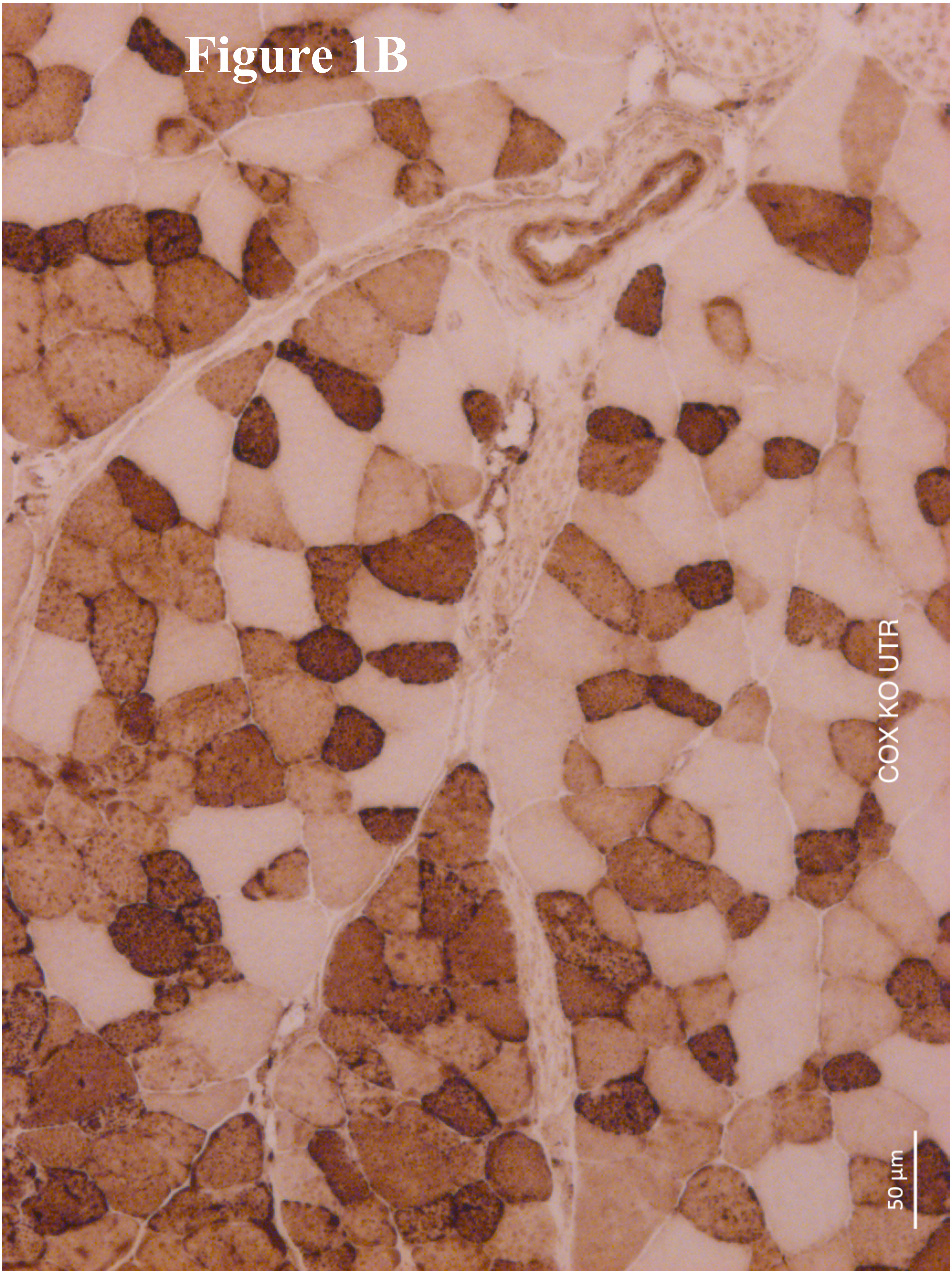

Figure 1B

Figure 1B

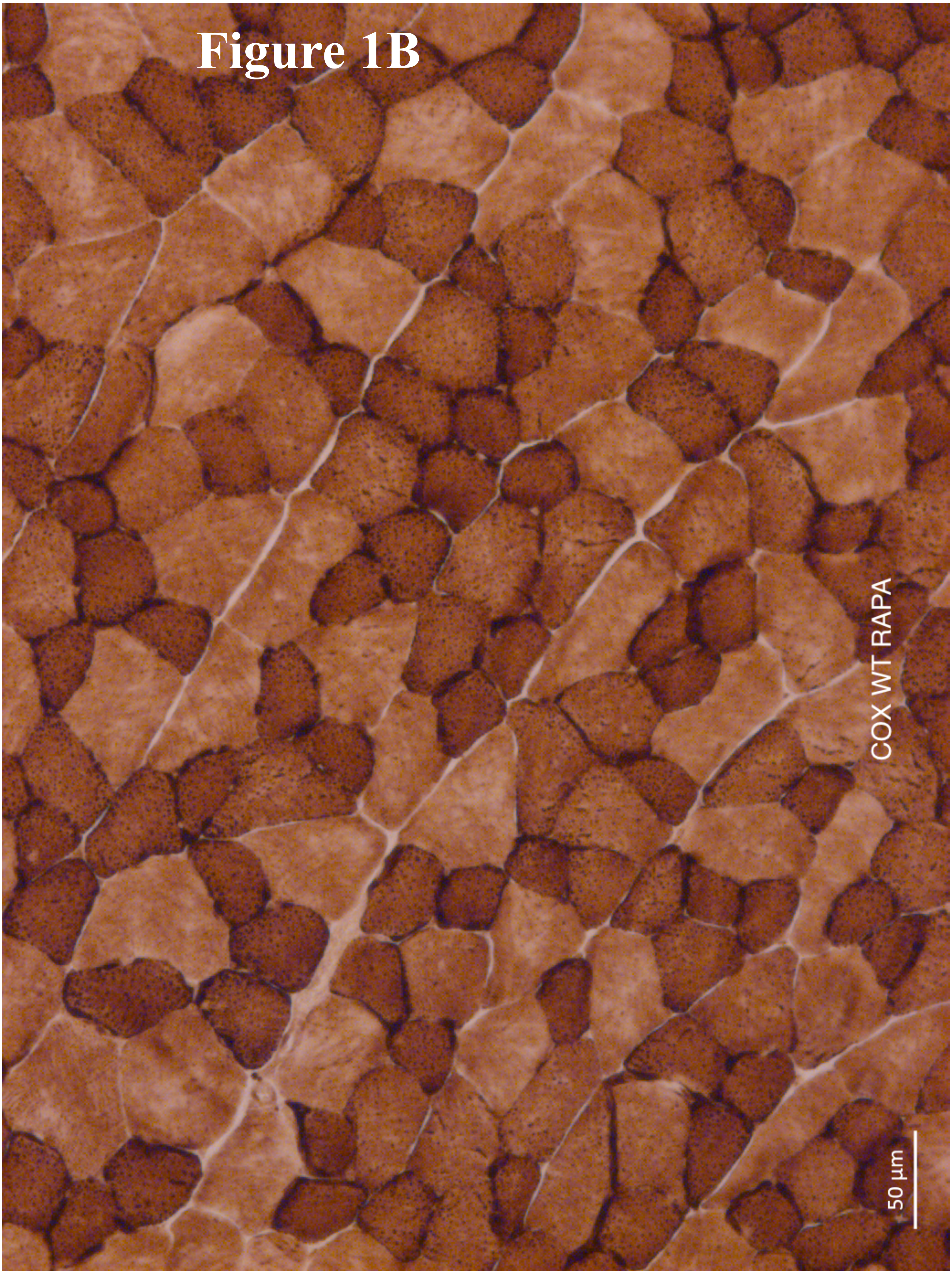

Figure 1B

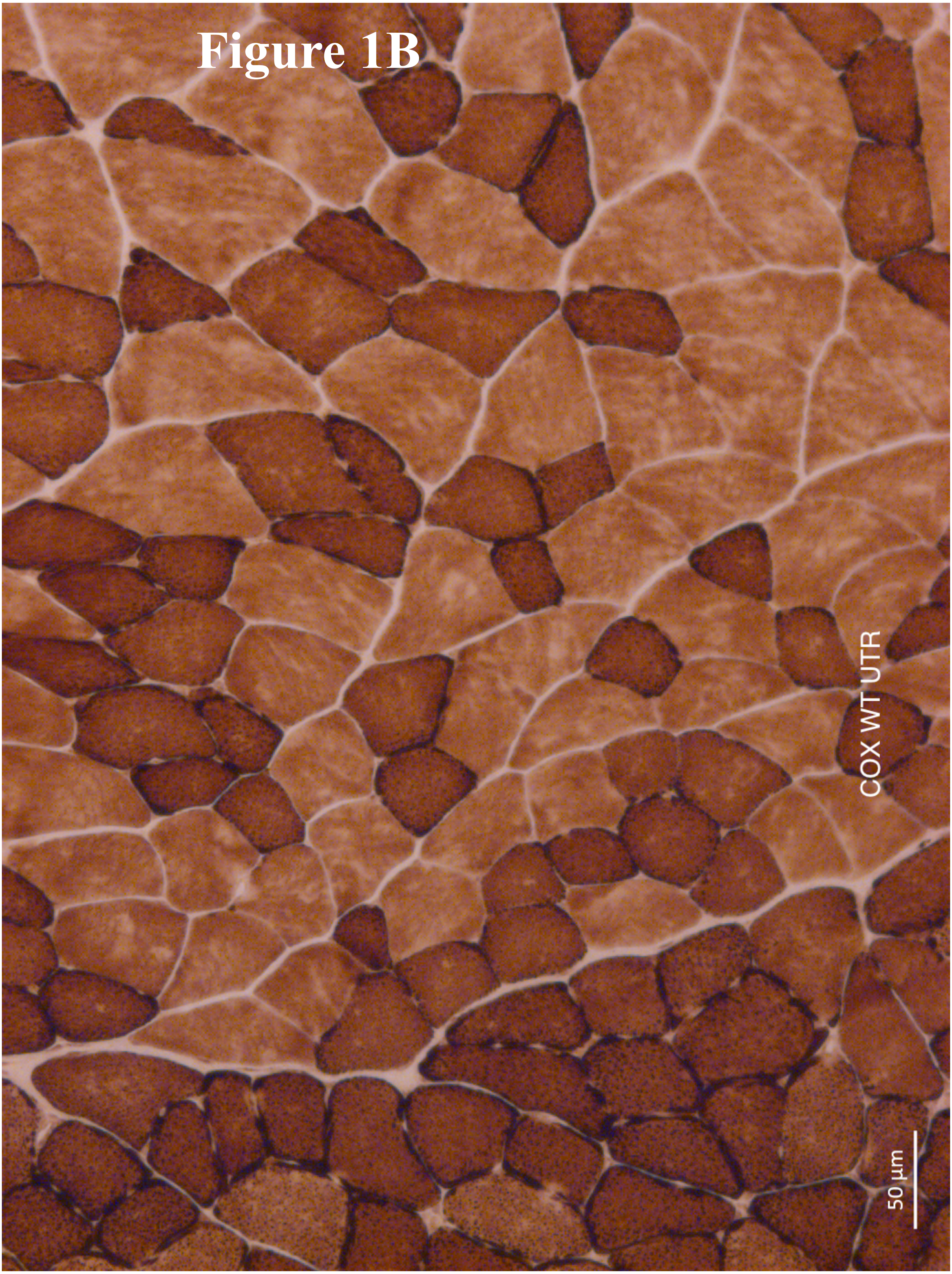

Figure 1B

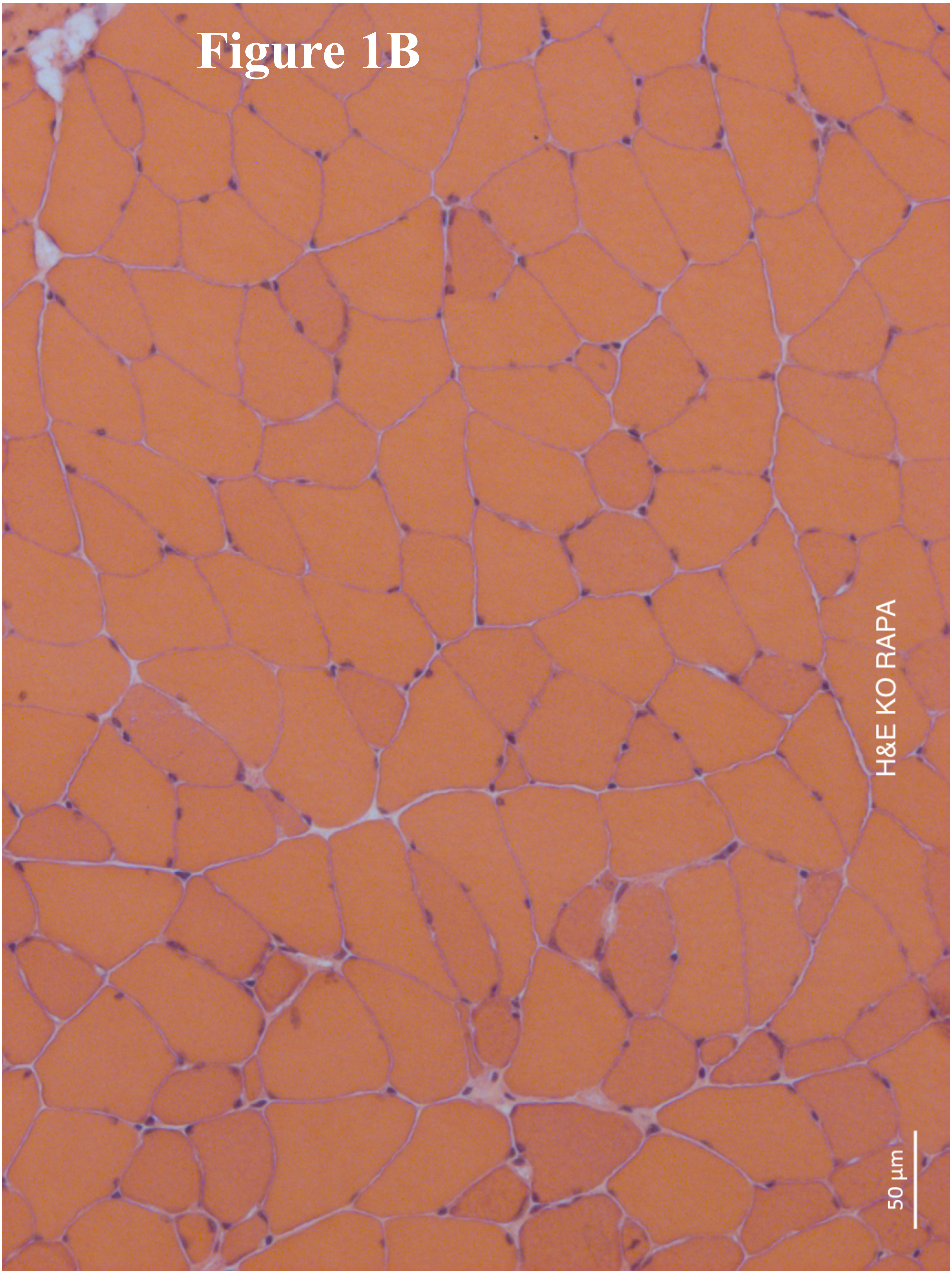

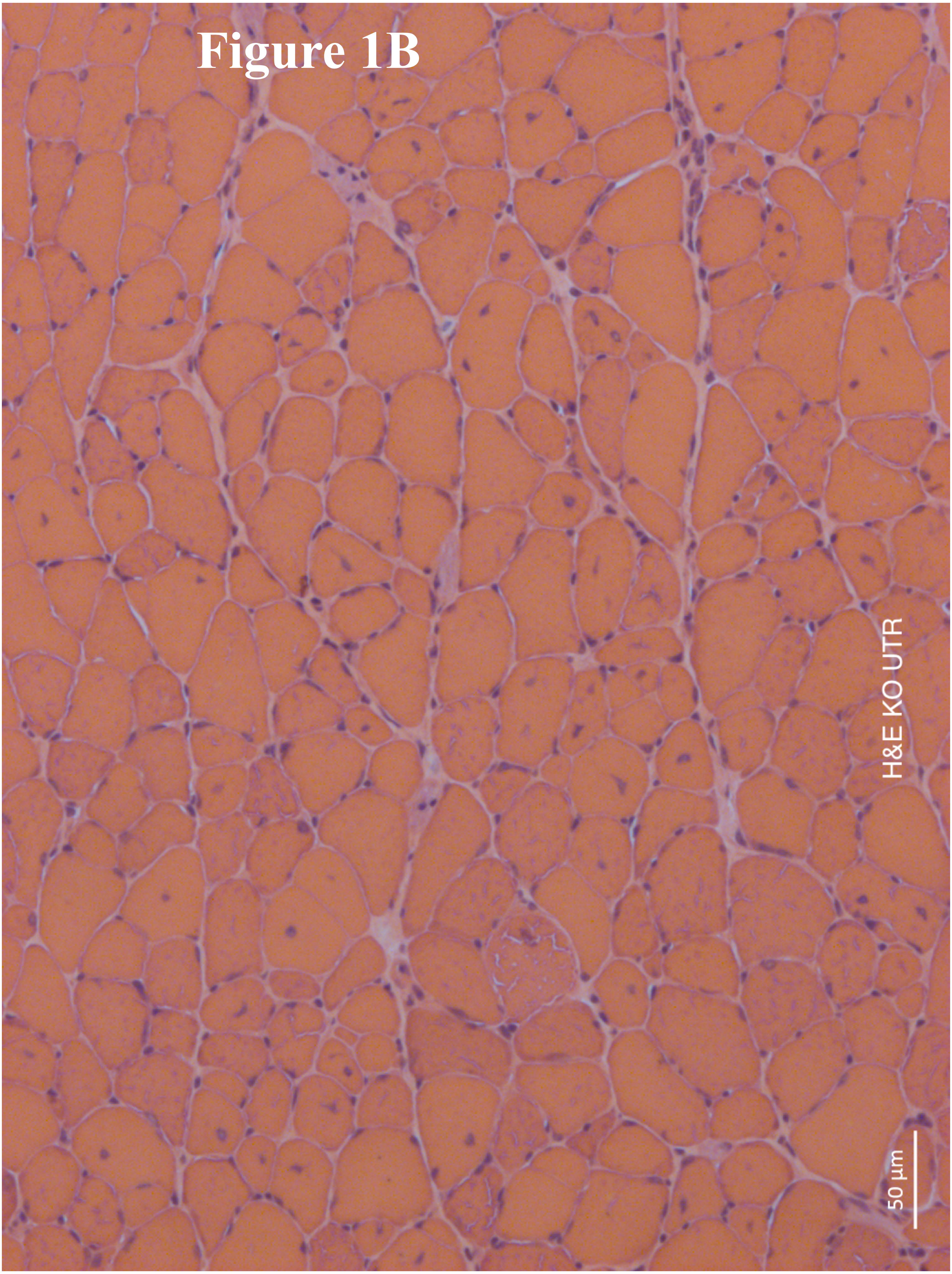

Figure 1B

Figure 1B

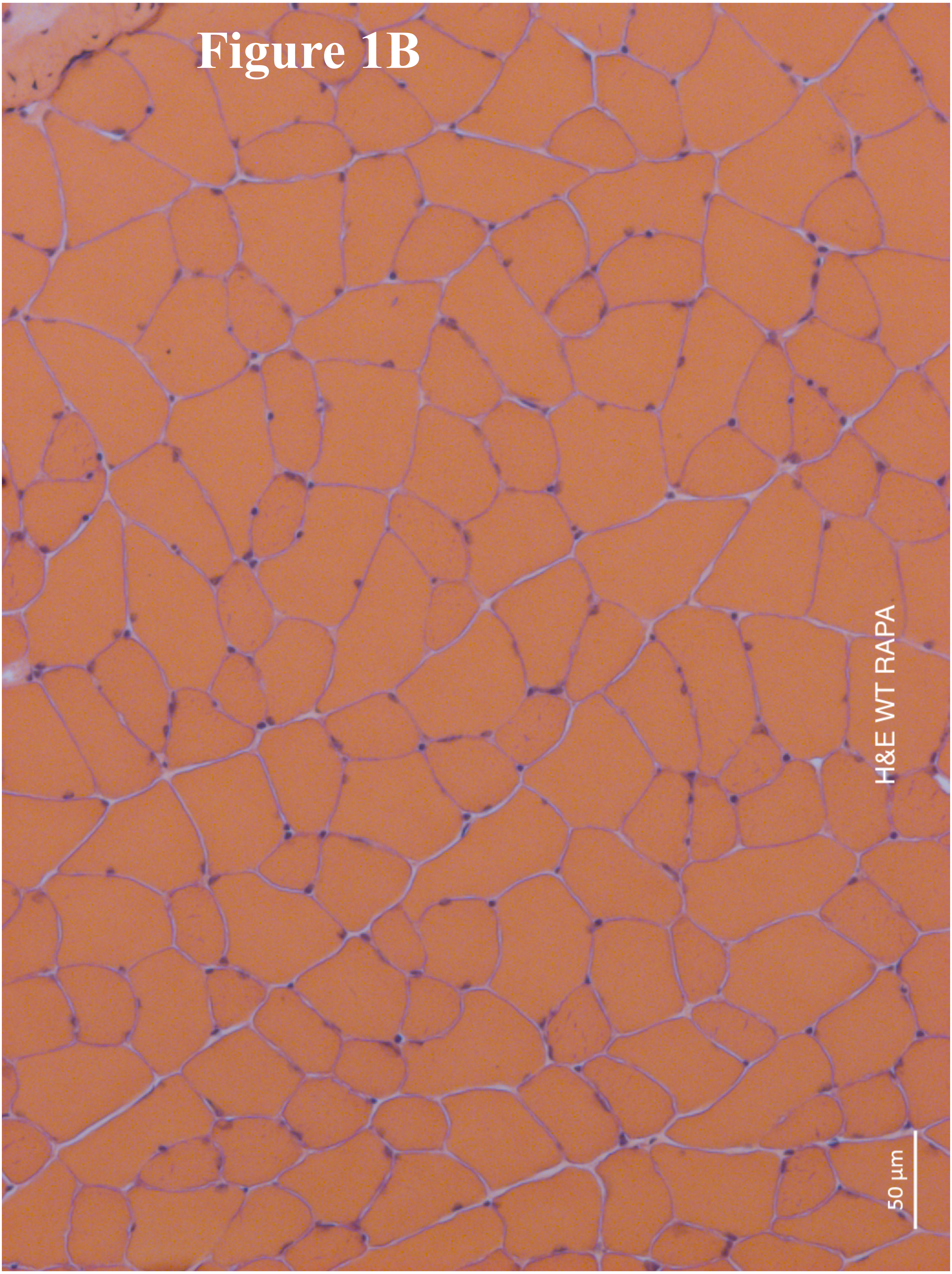

Figure 1B

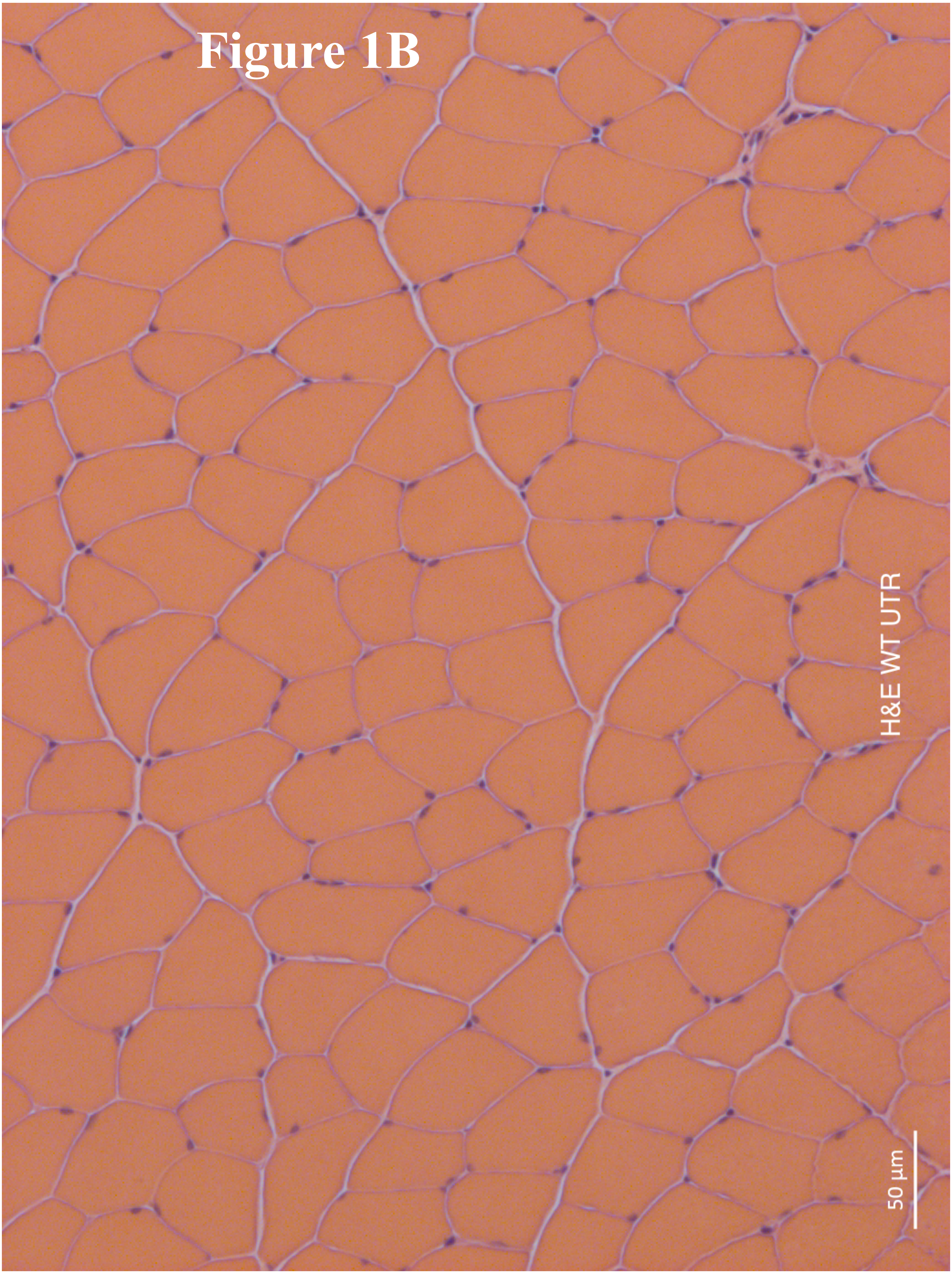

Figure 1B

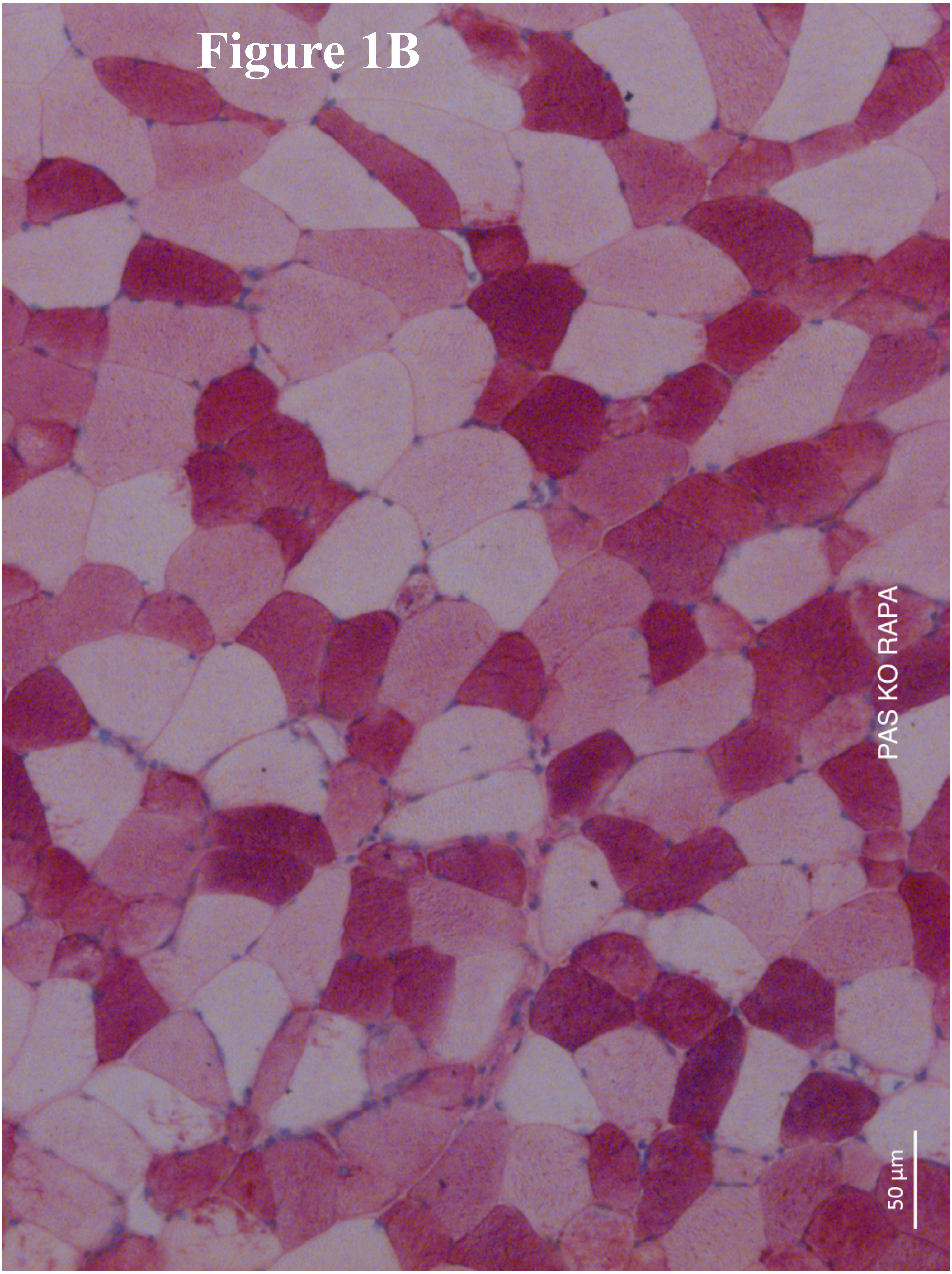

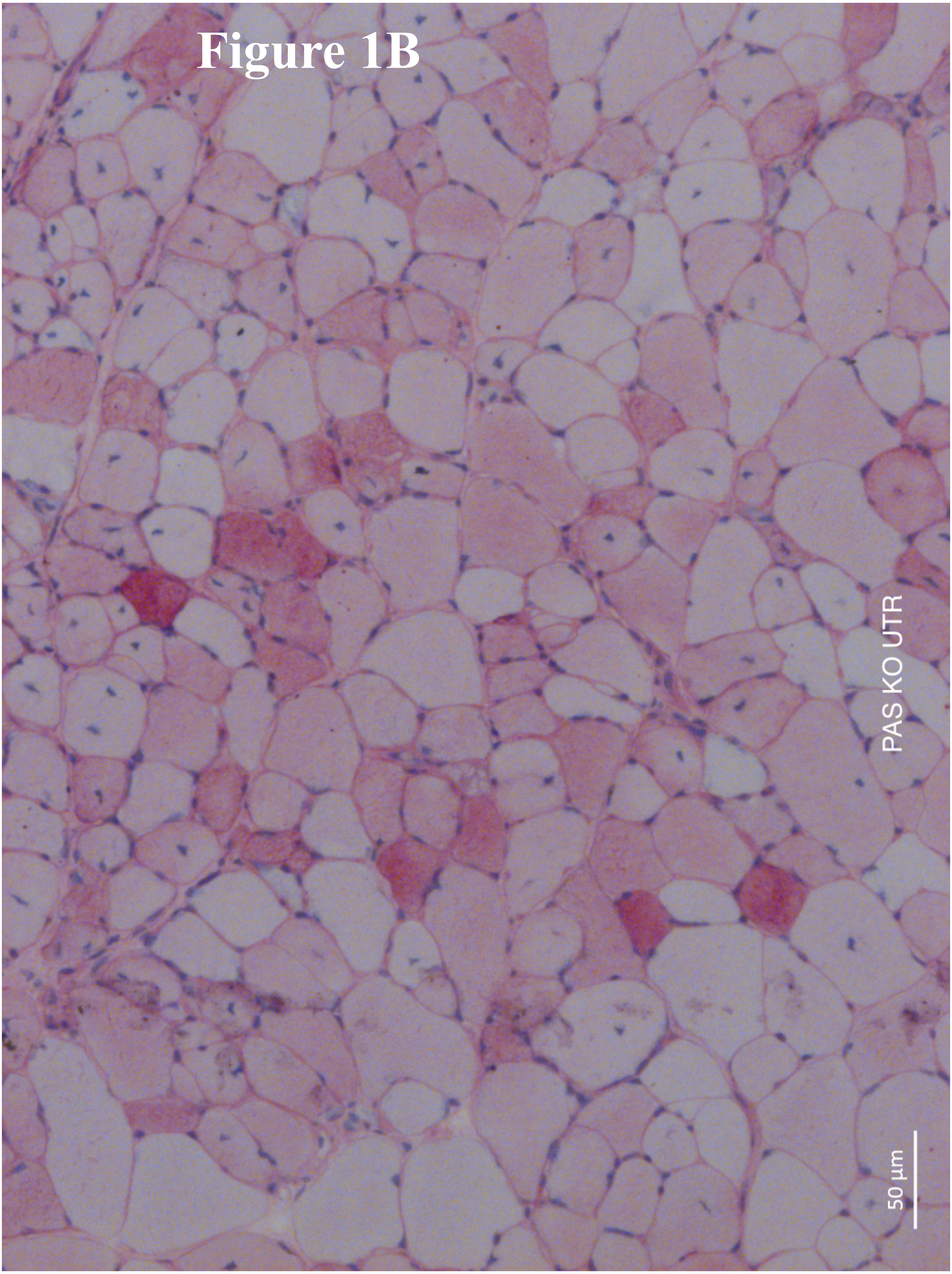
Figure 1B
PAS KO UTR
50 µm

Figure 1B

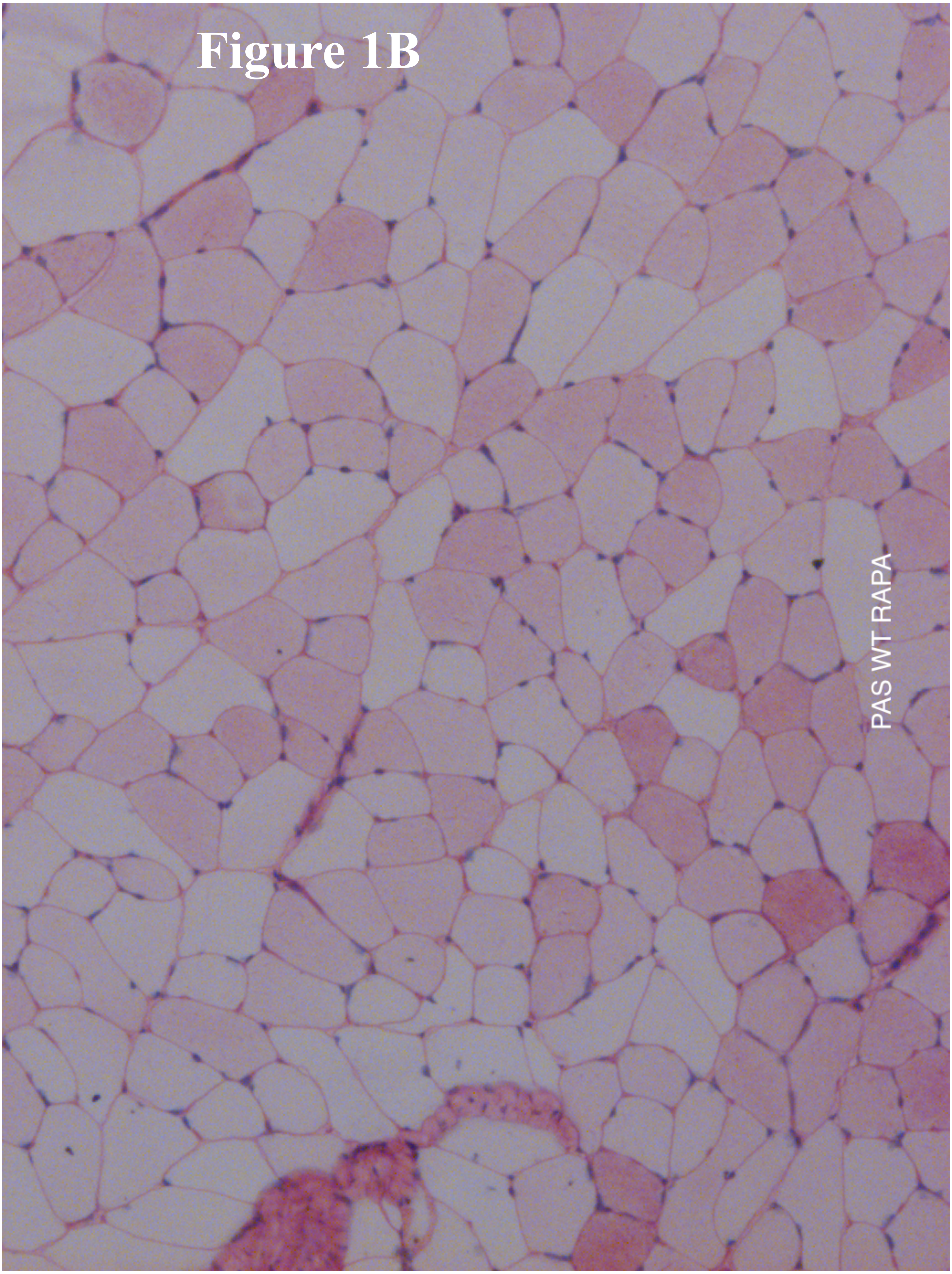

Figure 1B

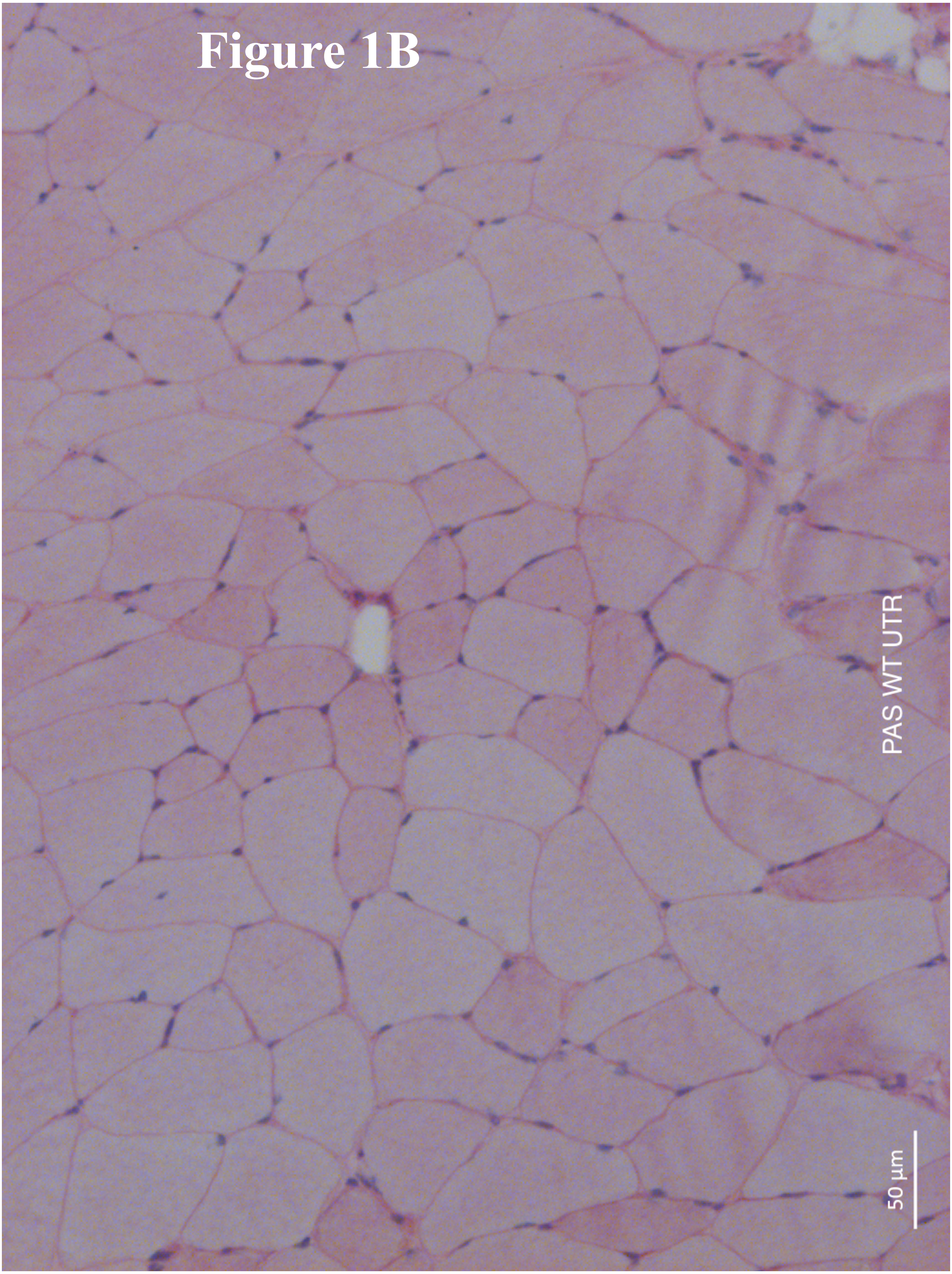

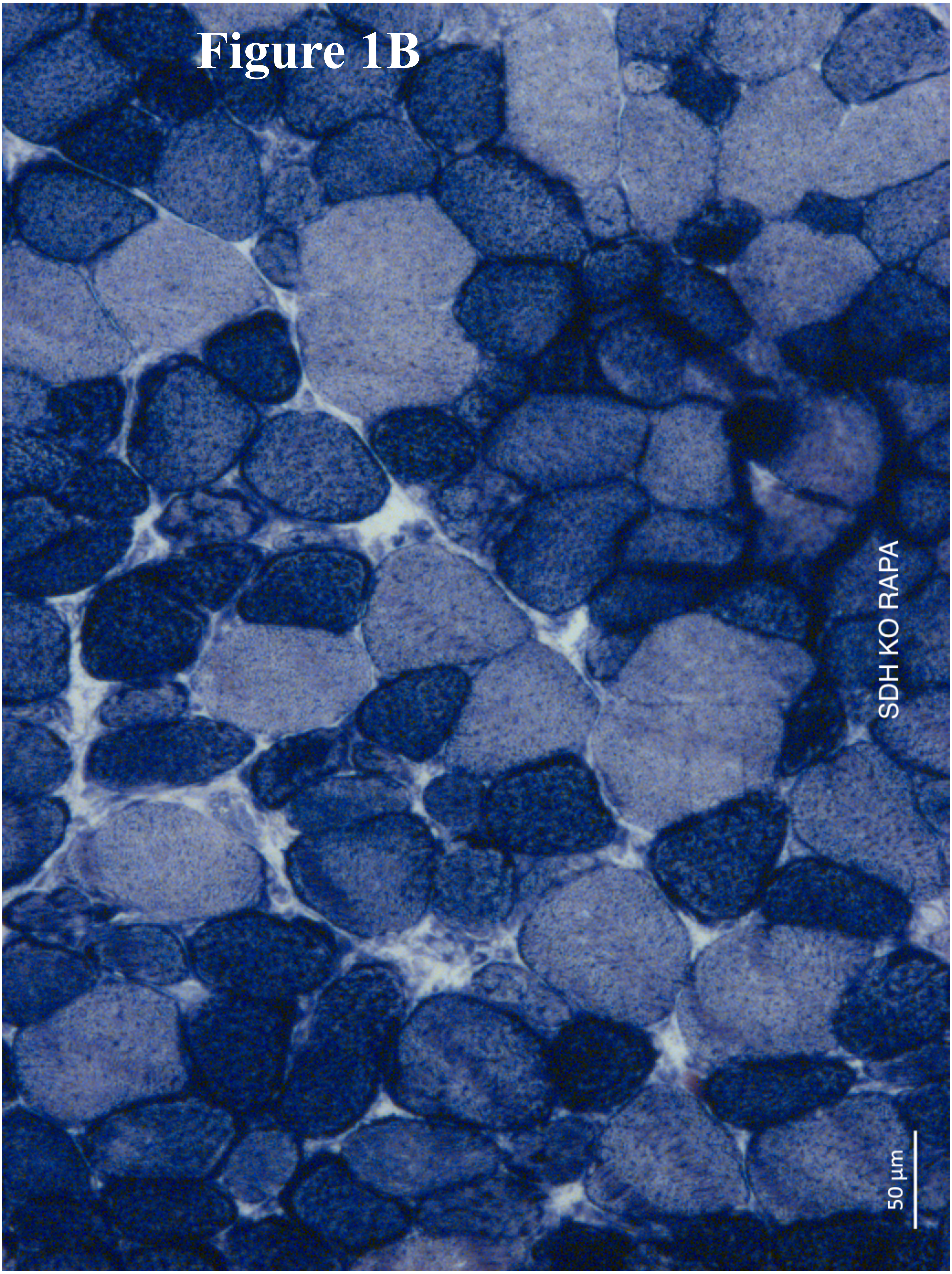
Figure 1B
SDH KO RAPA
50 µm

Figure 1B

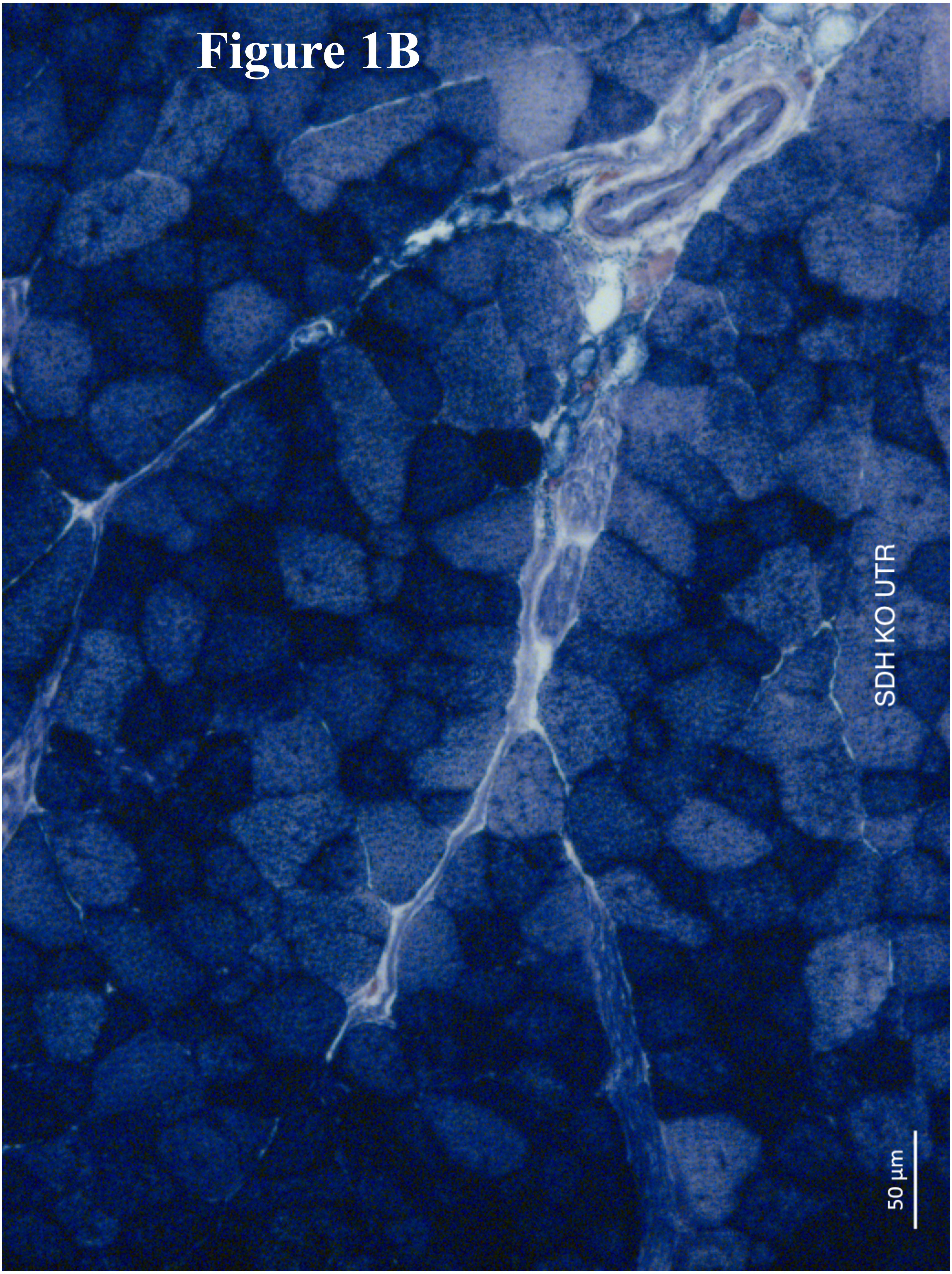

Figure 1B

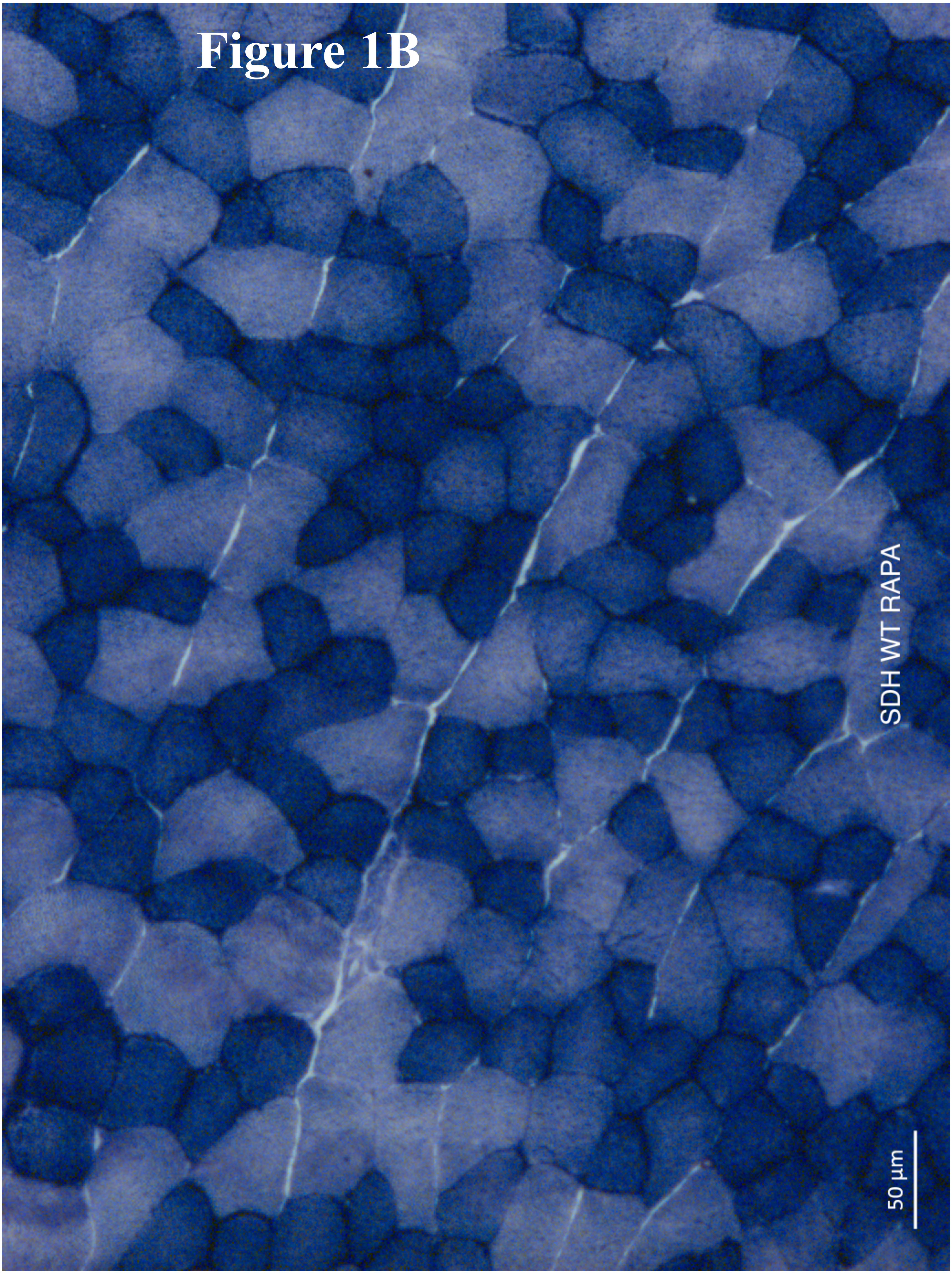

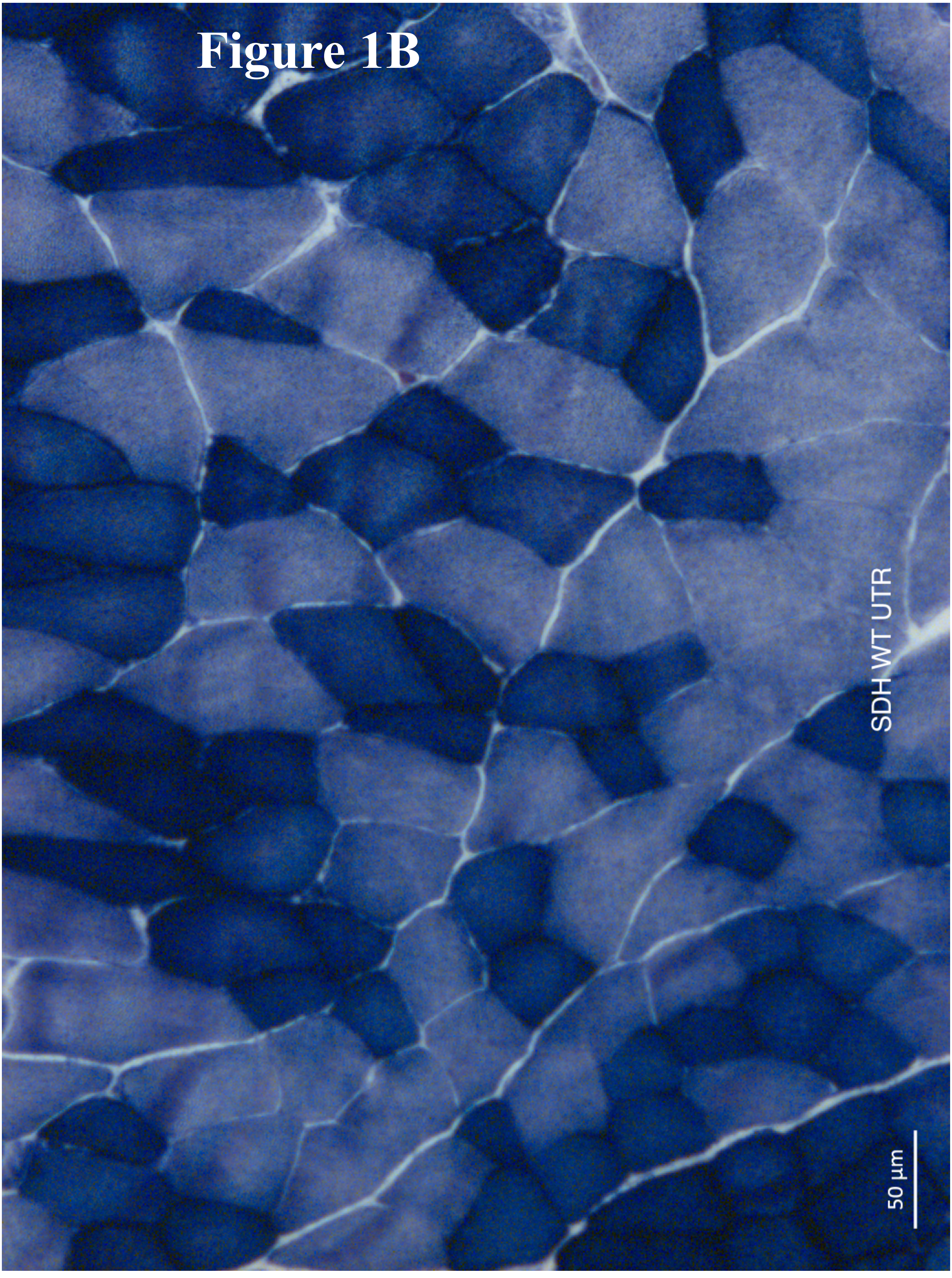

Figure 1B
SDH WT UTR
50 µm
