# Supplementary material for: Rapamycin rescues mitochondrial myopathy via coordinated activation of autophagy and lysosomal biogenesis
Source: EMBO Mol Med. 2018 Oct 11;10(11):e8799. doi: 10.15252/emmm.201708799 (PMC6220341; doi:10.15252/emmm.201708799)

Figure 5A

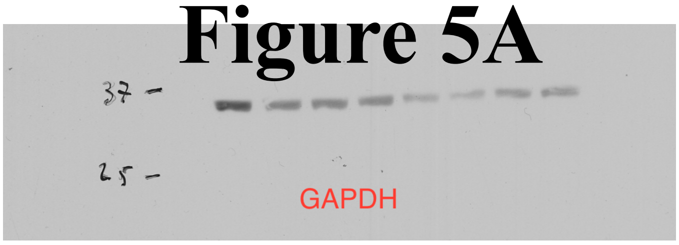

Figure 5A

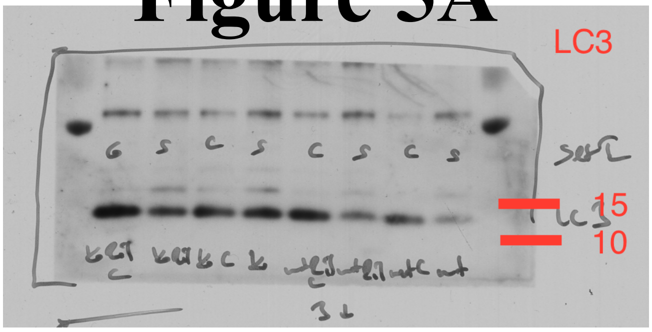

Figure 5C

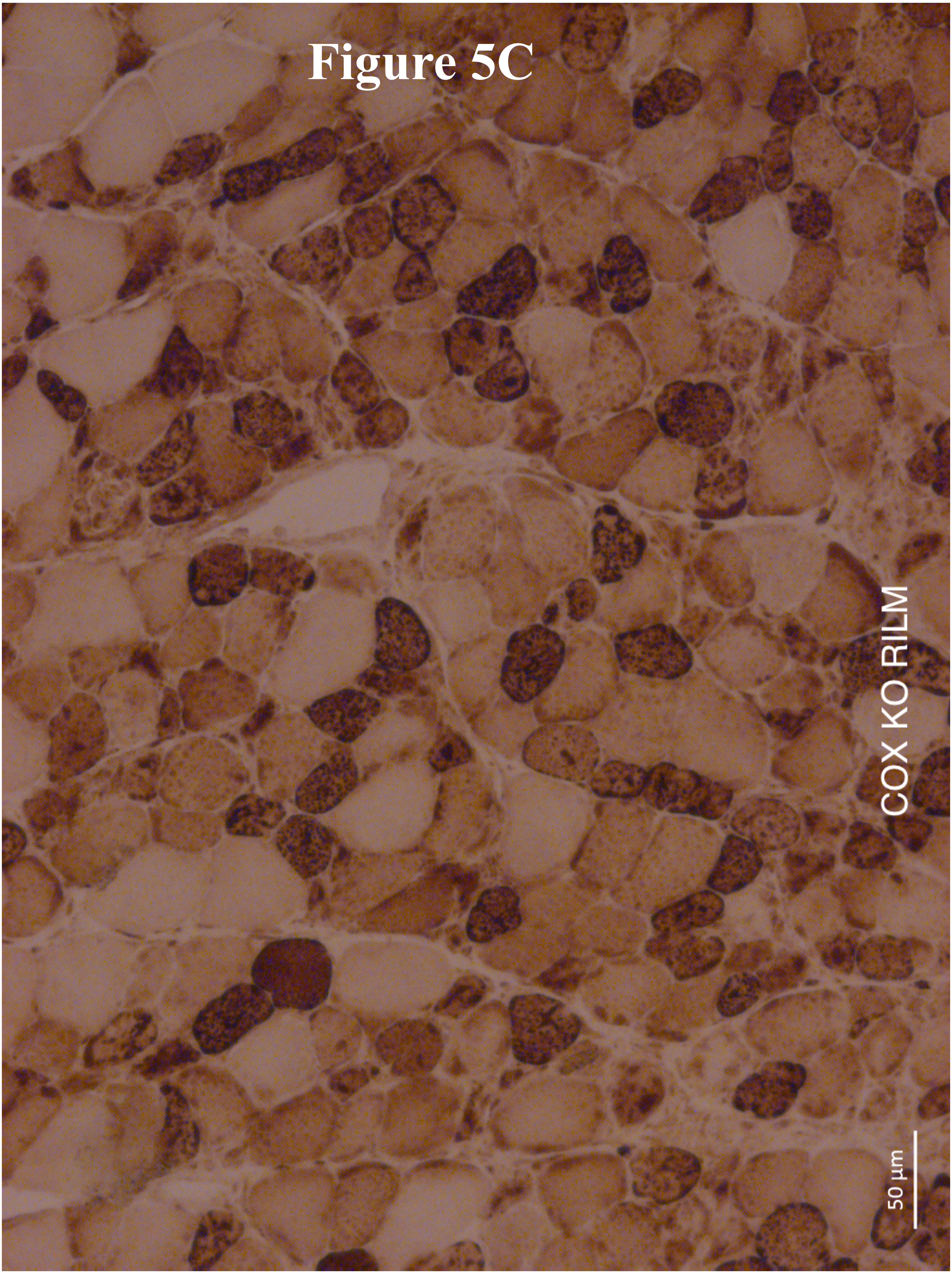

Figure 5C

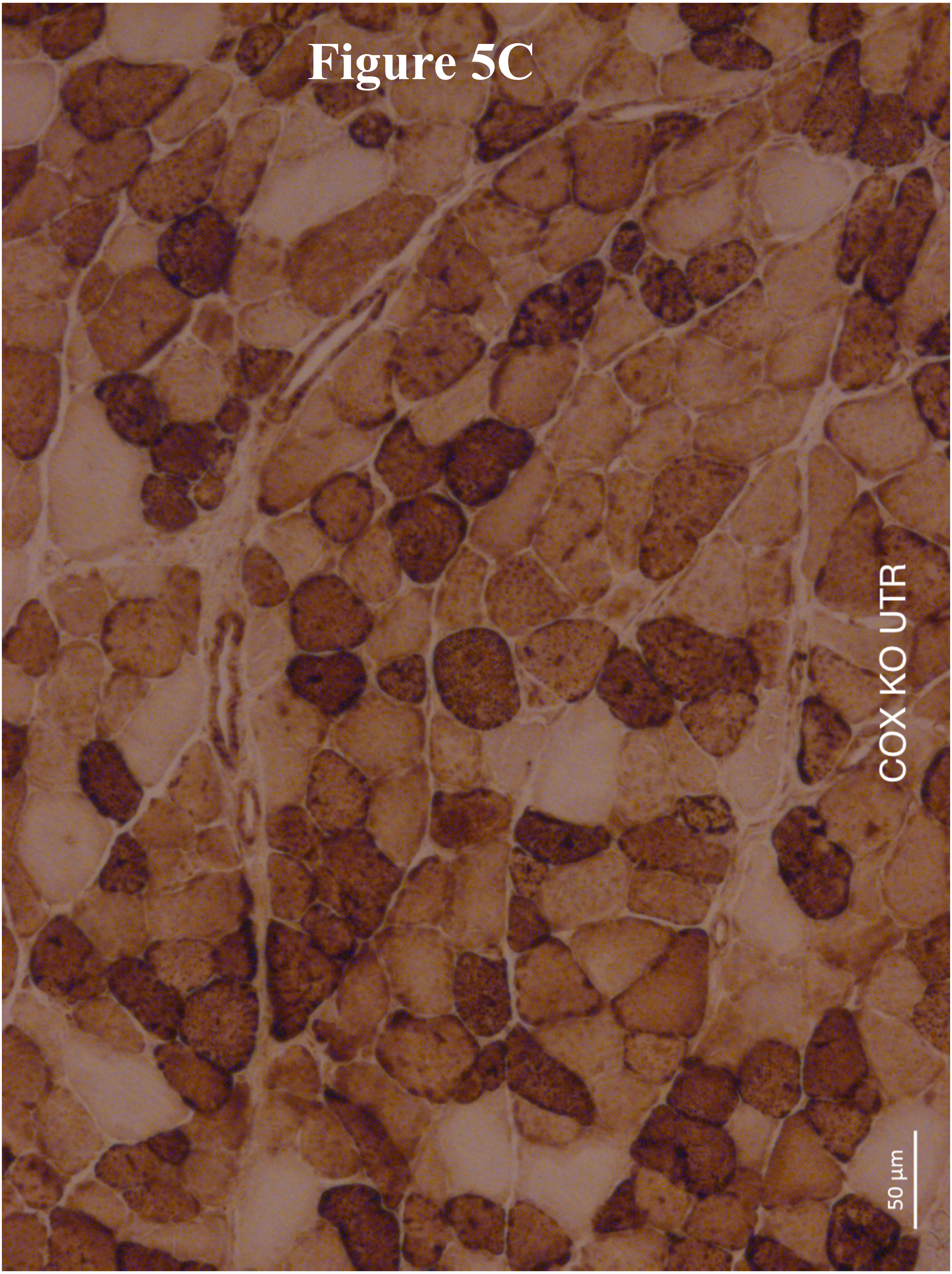

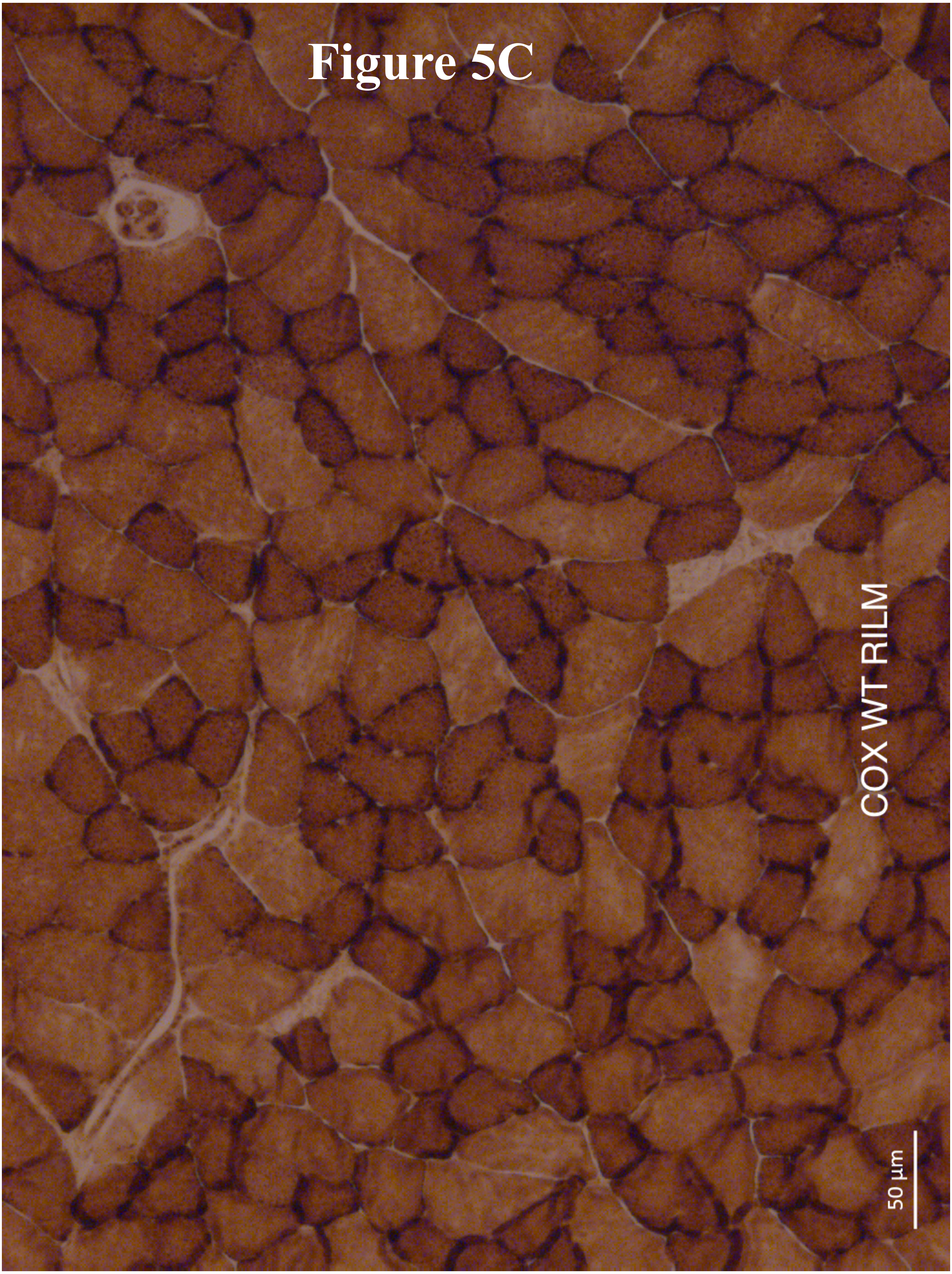
Figure 5C
COX WT RILM
50 µm

Figure 5C

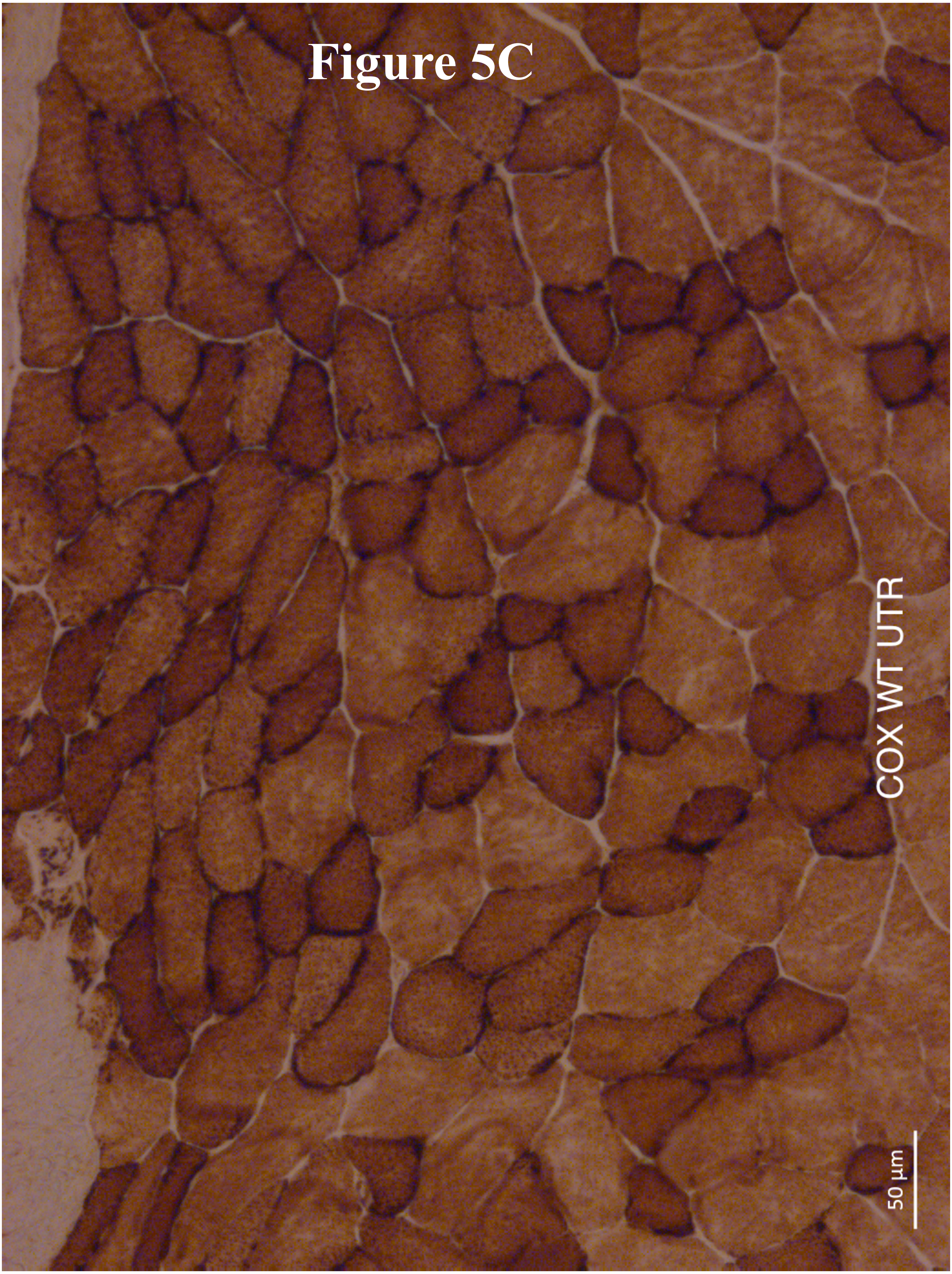

Figure 5C

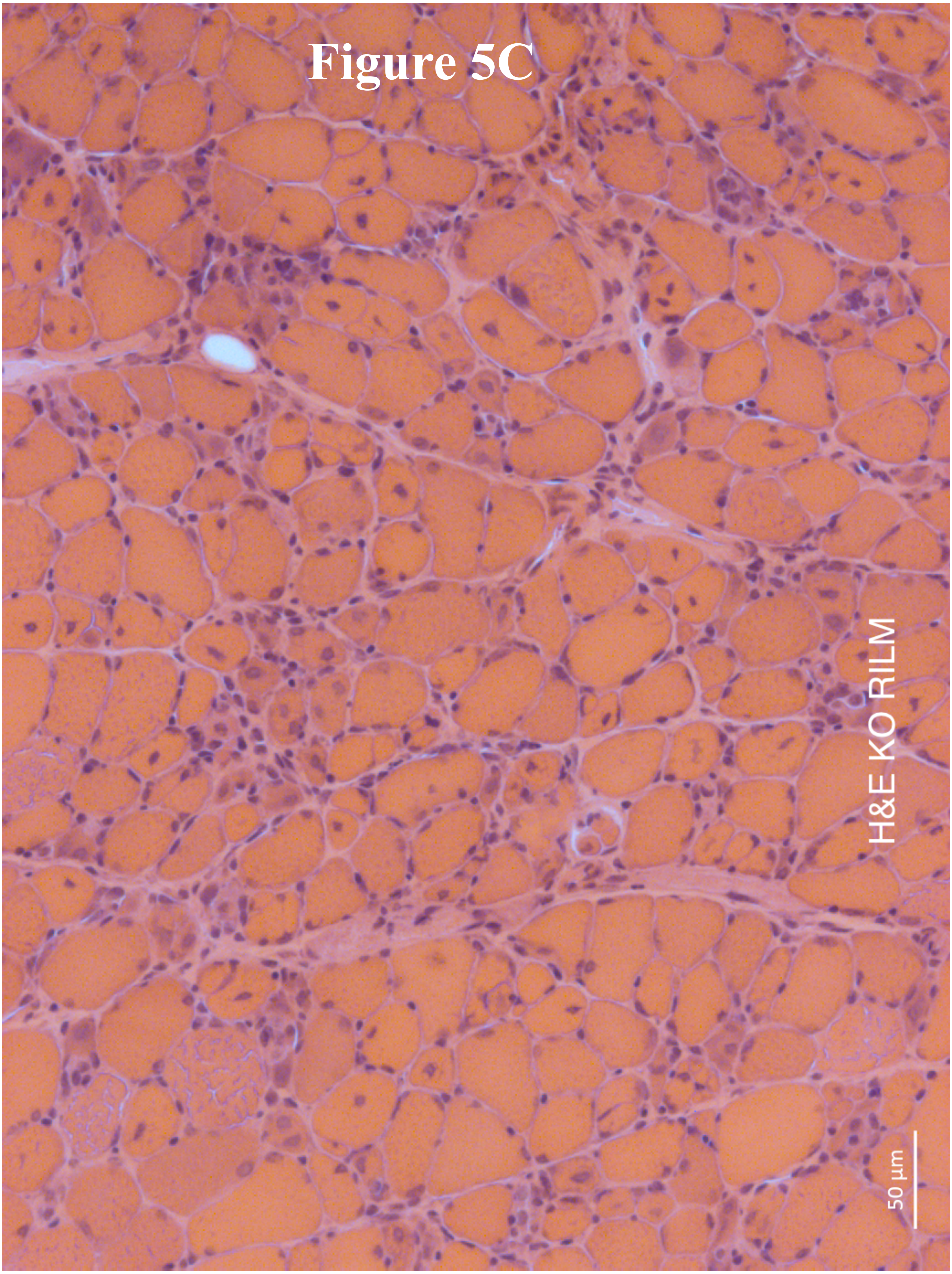

Figure 5C

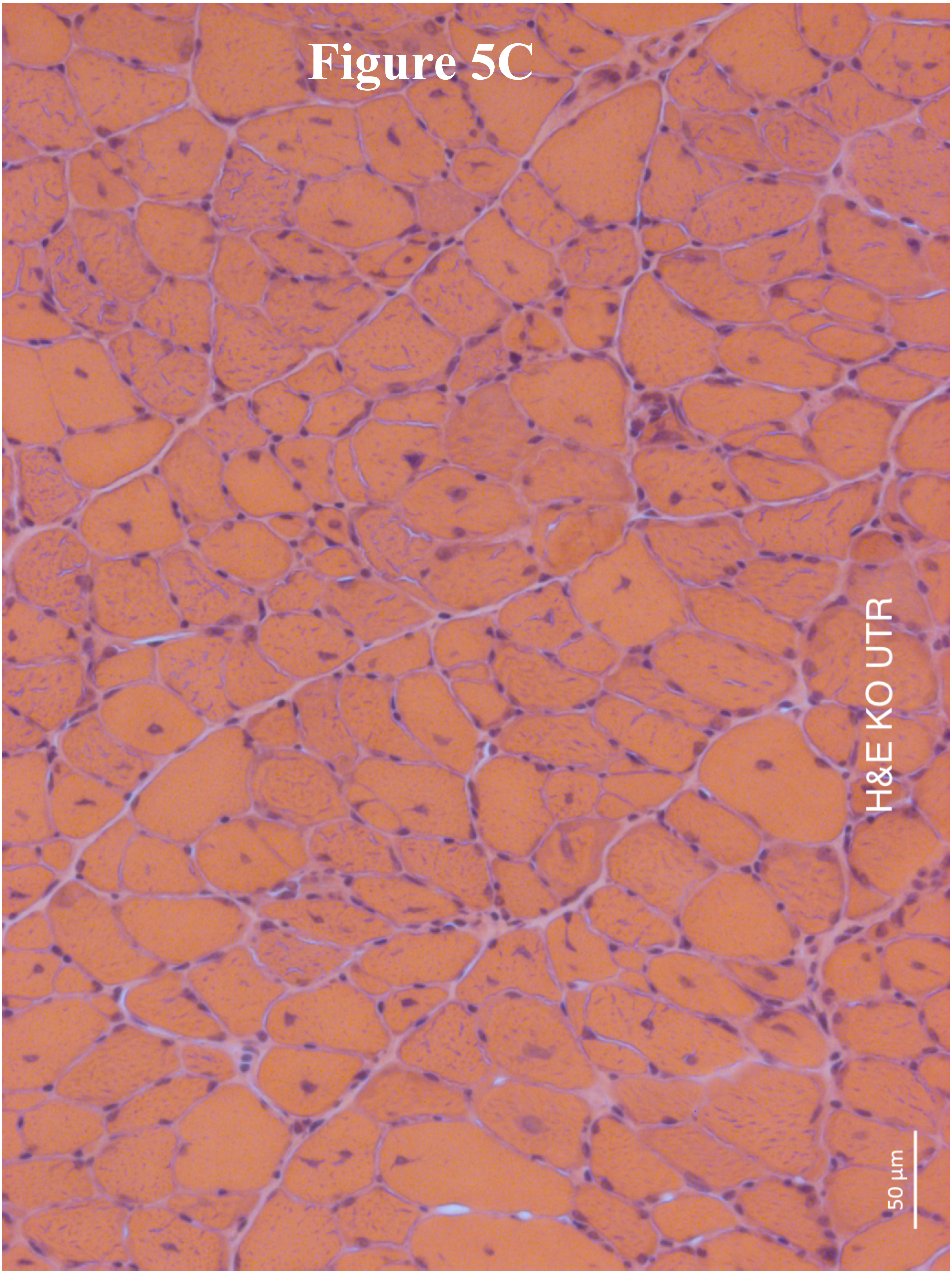

Figure 5C

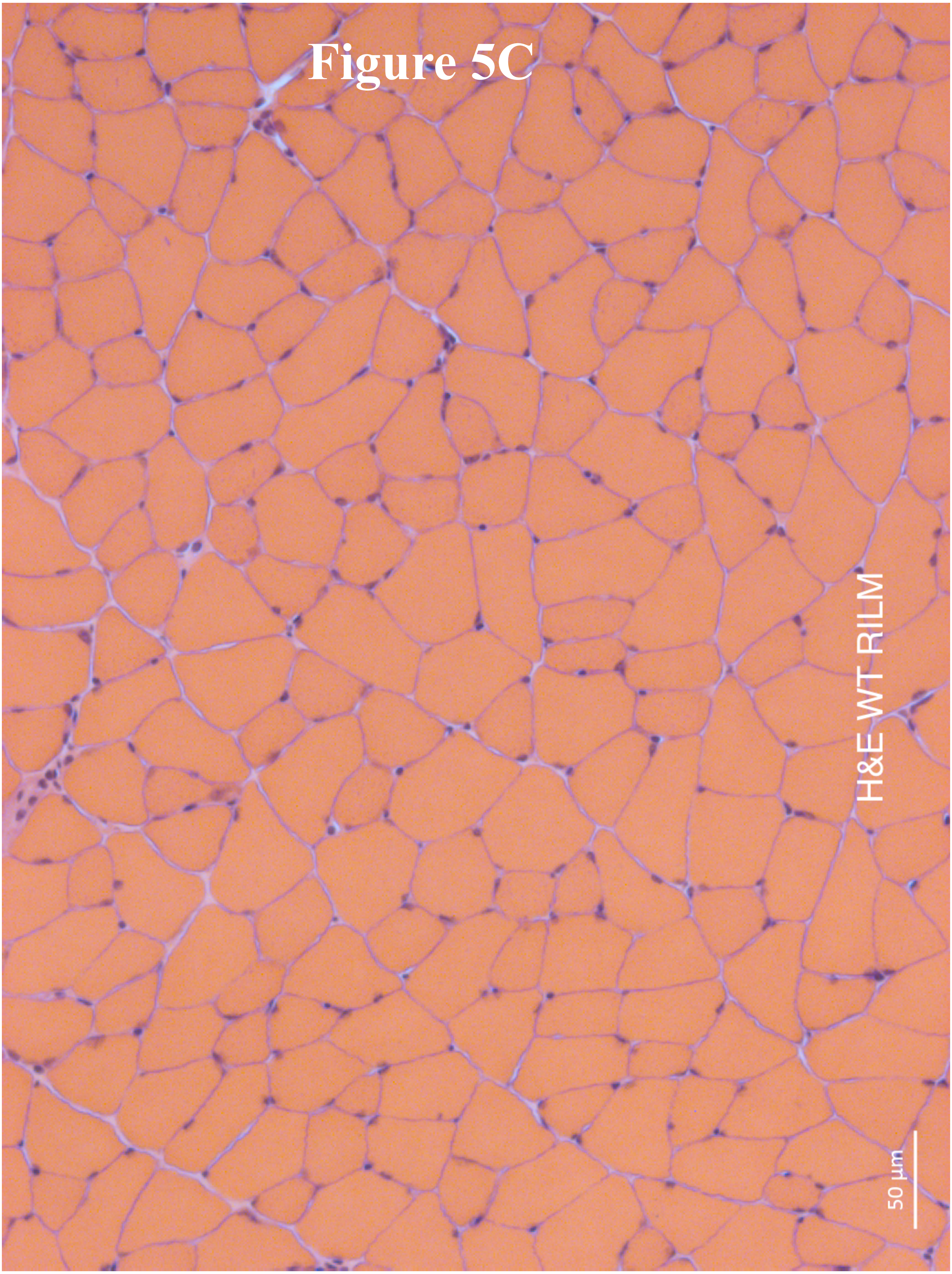

Figure 5C

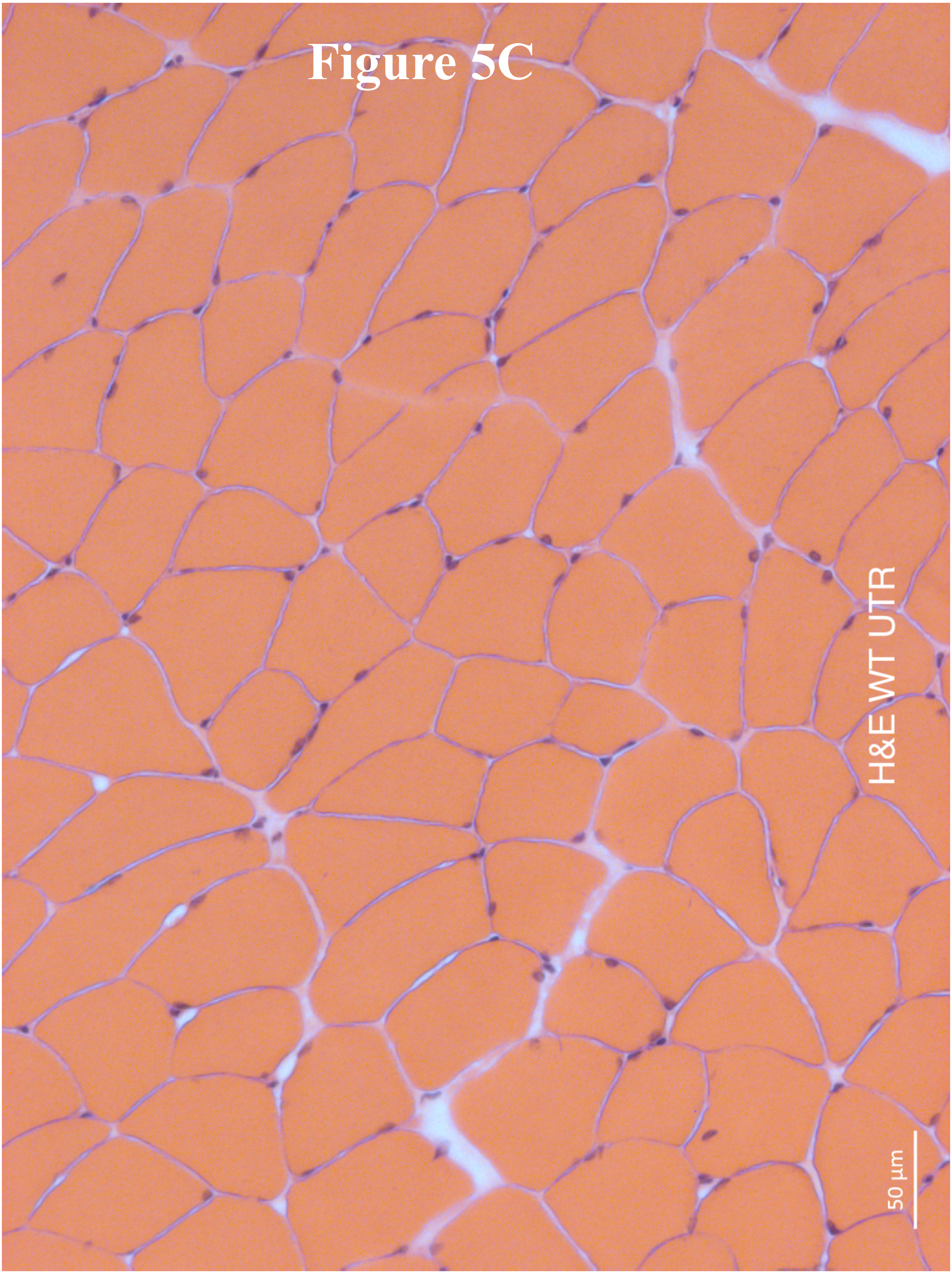

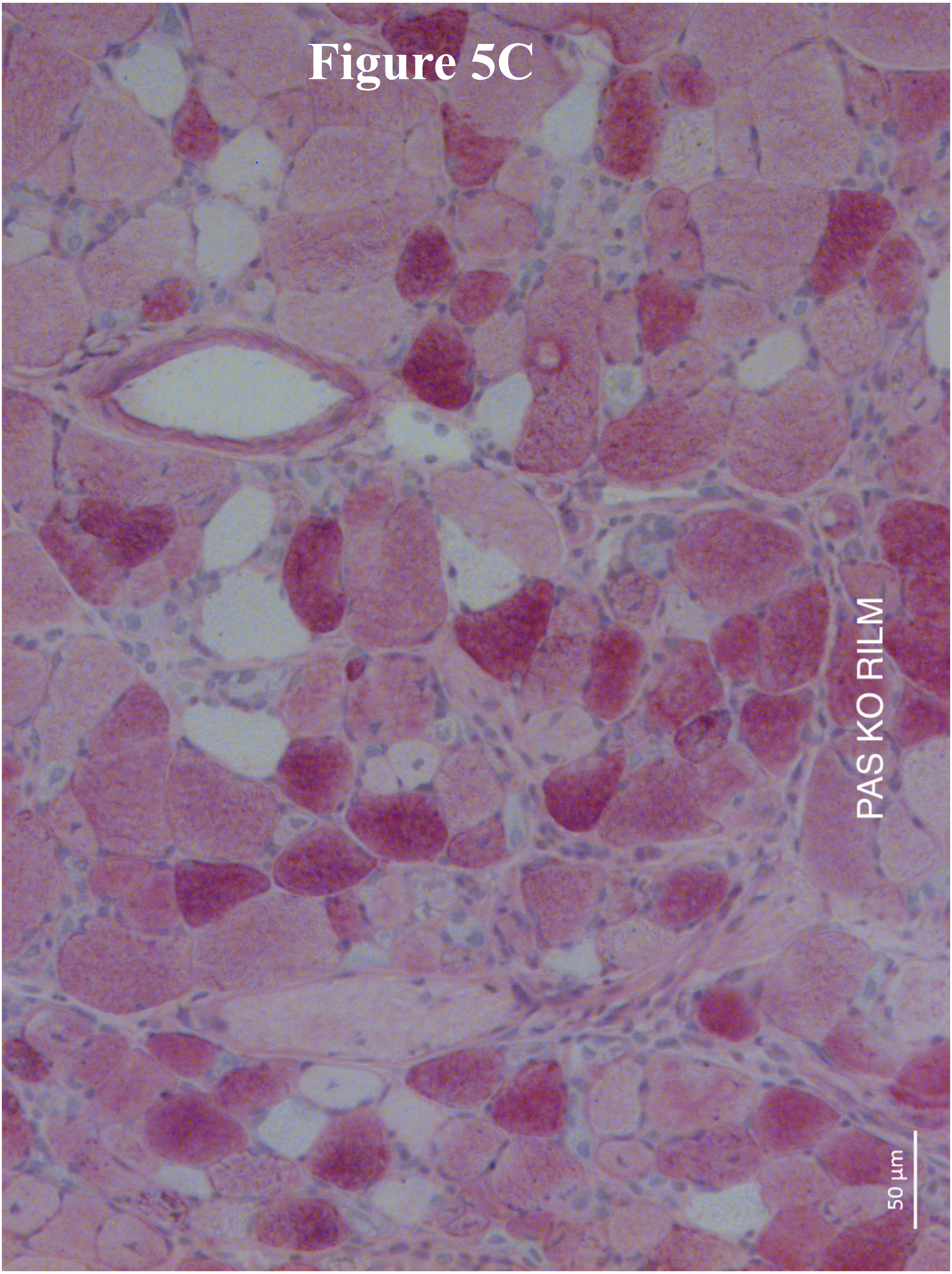

Figure 5C

Figure 5C

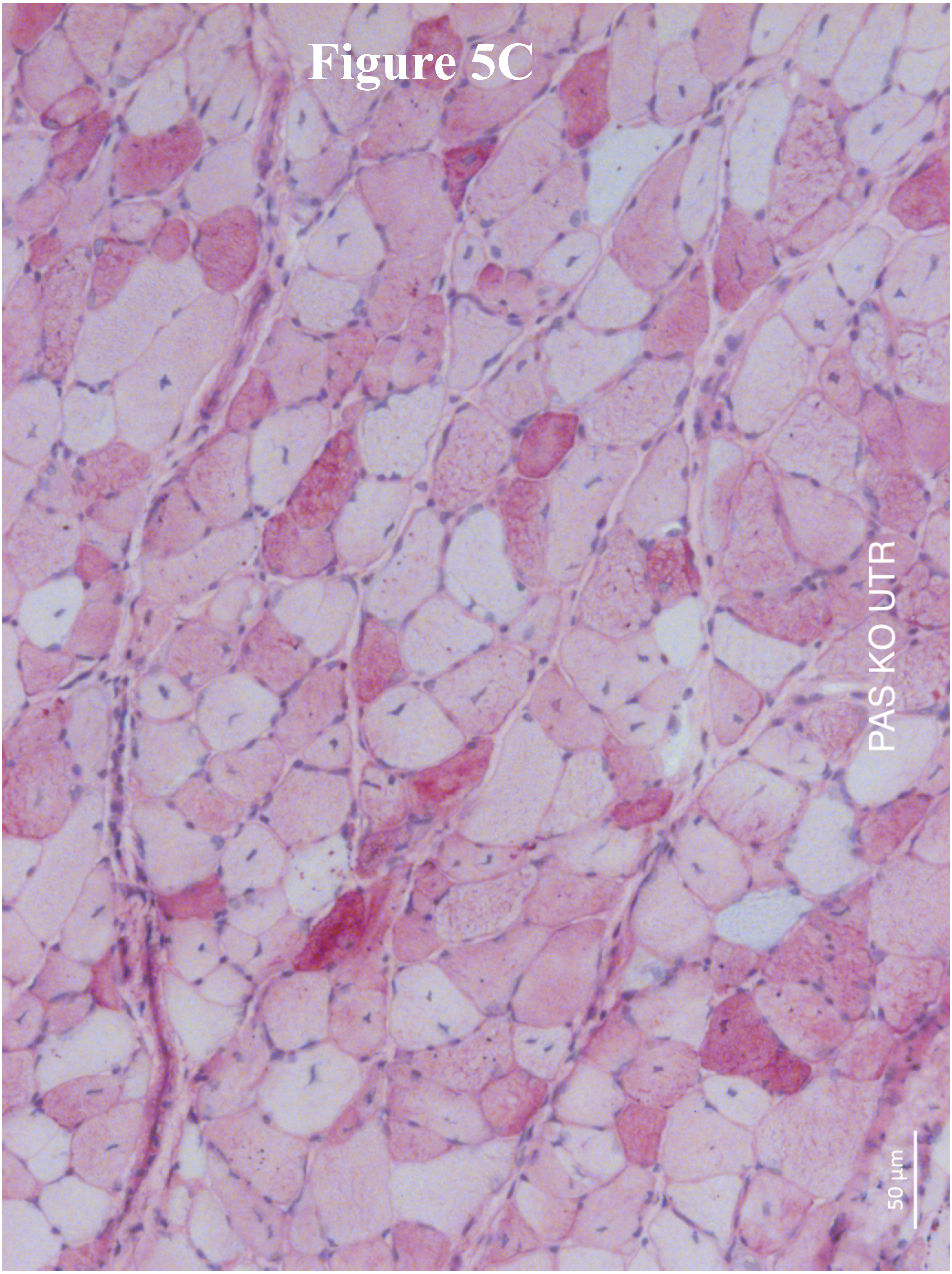

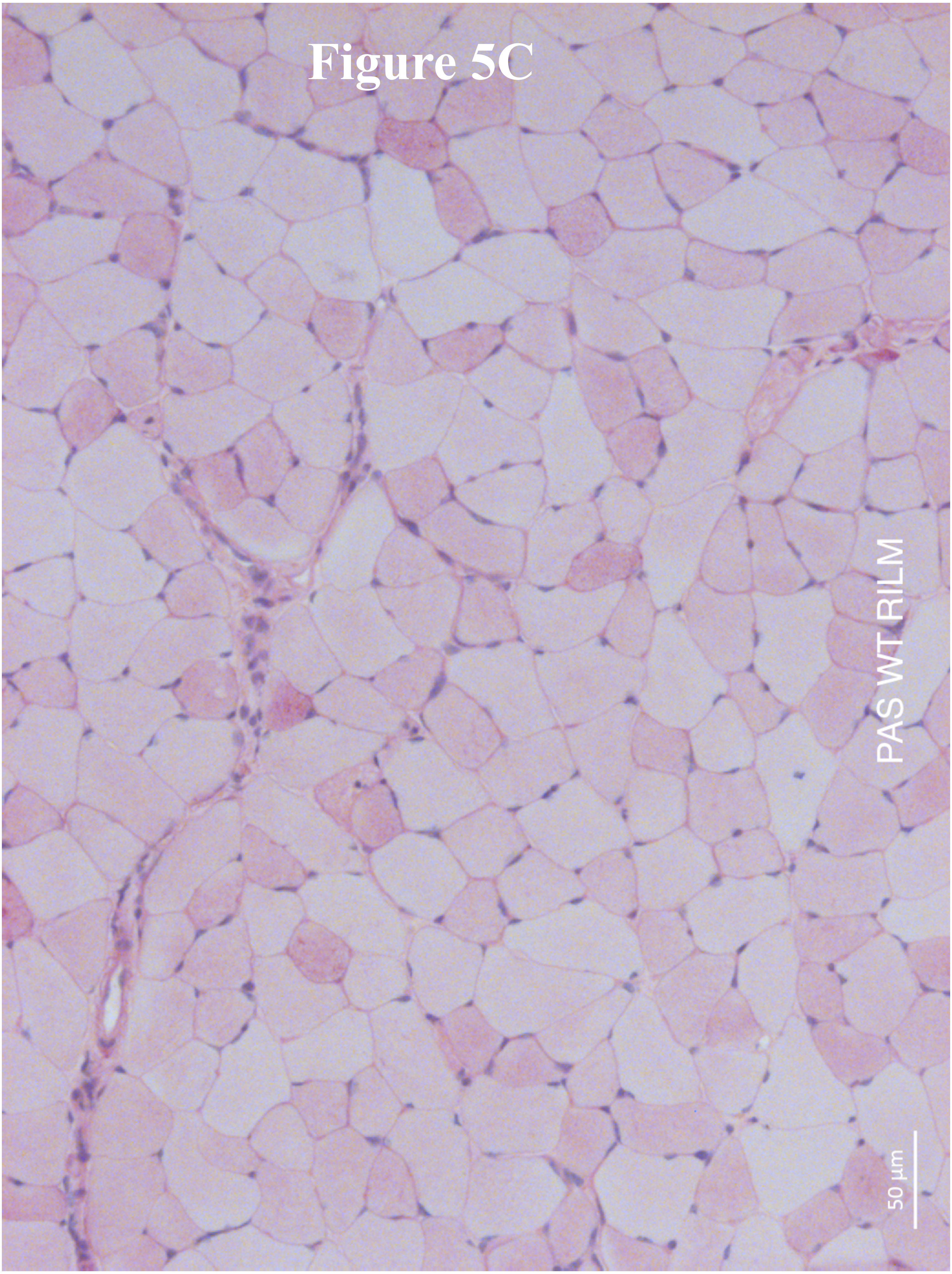
Figure 5C
PAS WT RILM
50 µm

Figure 5C

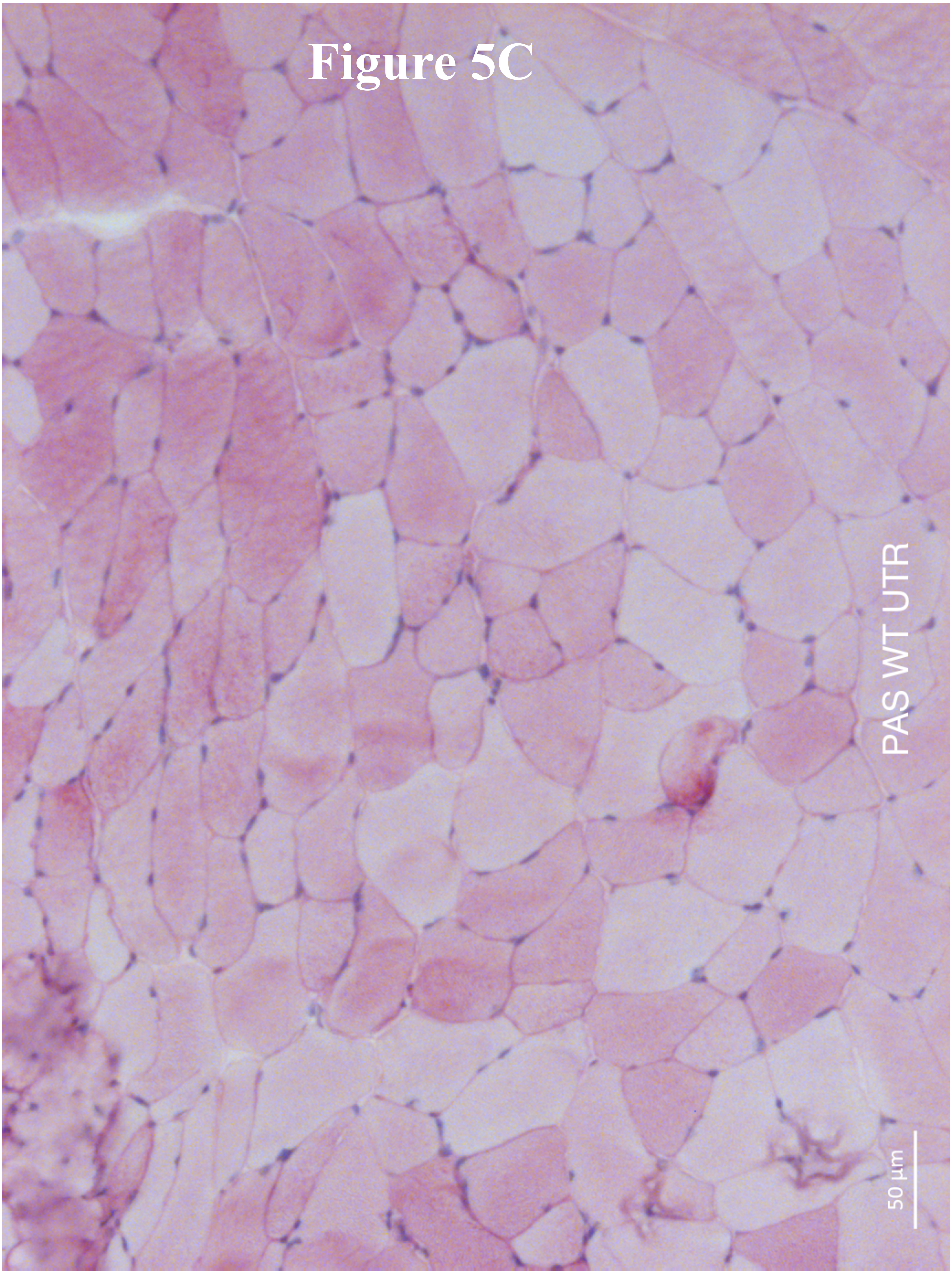

Figure 5C

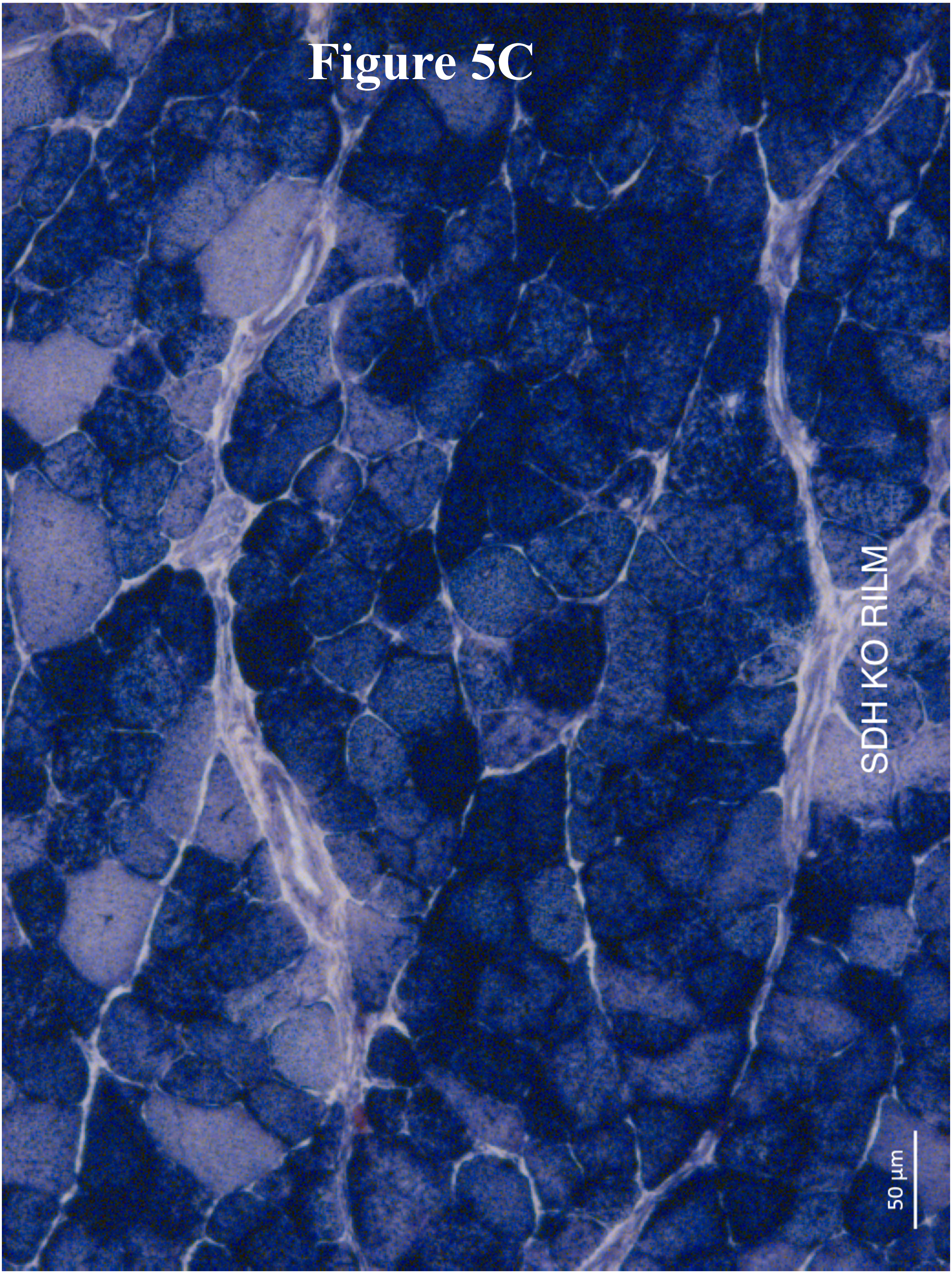

Figure 5C

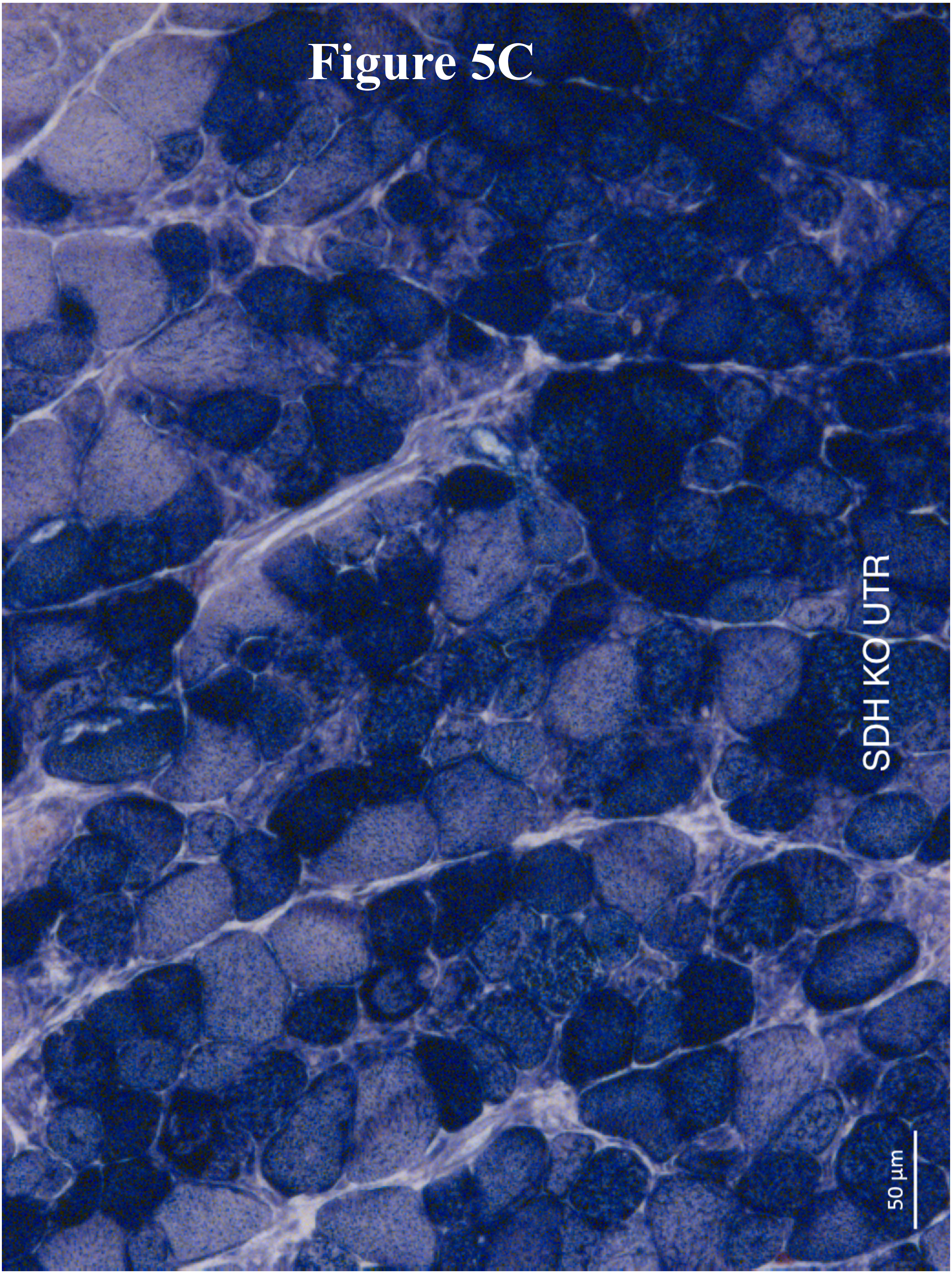

Figure 5C

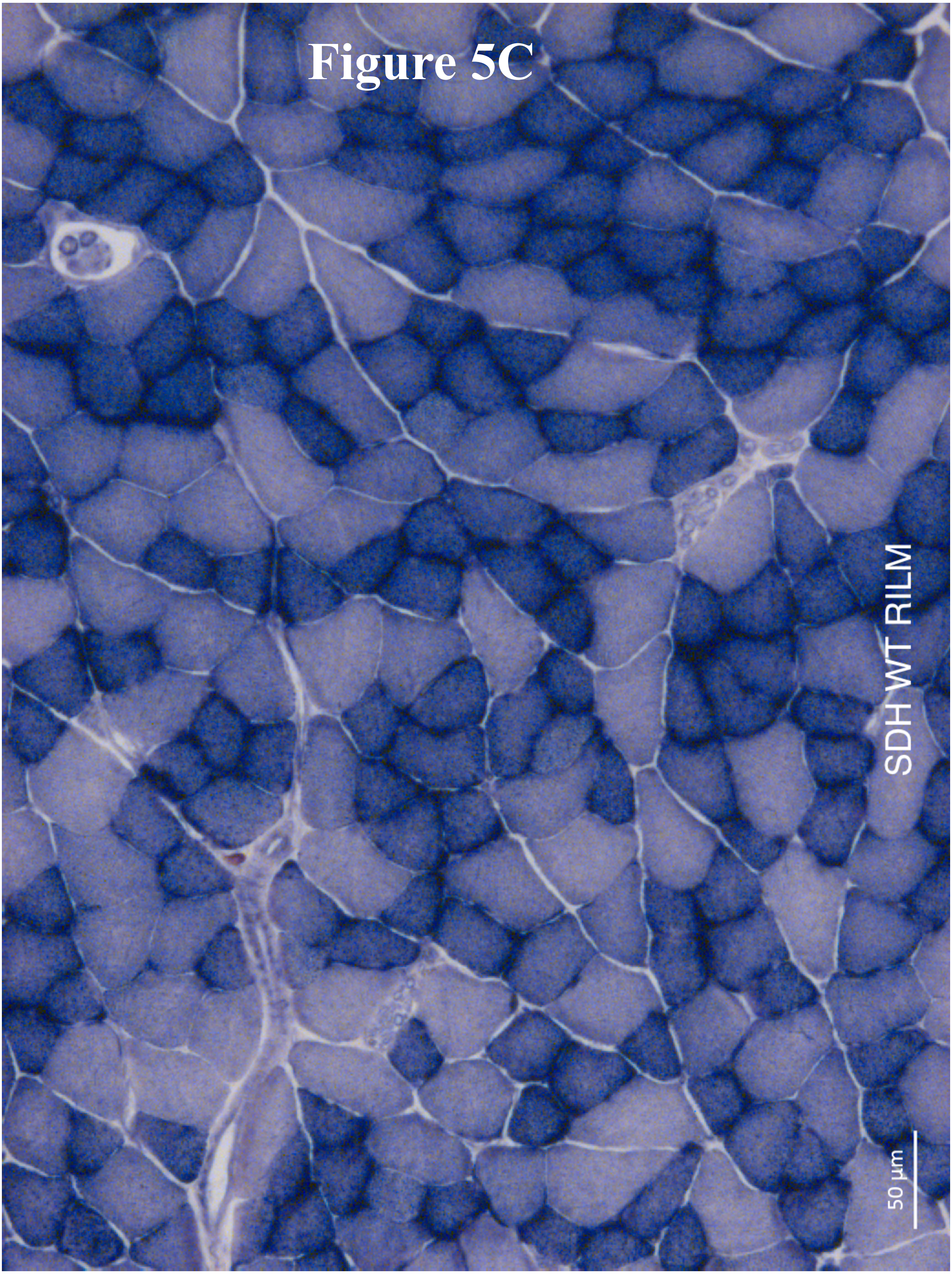

Figure 5C

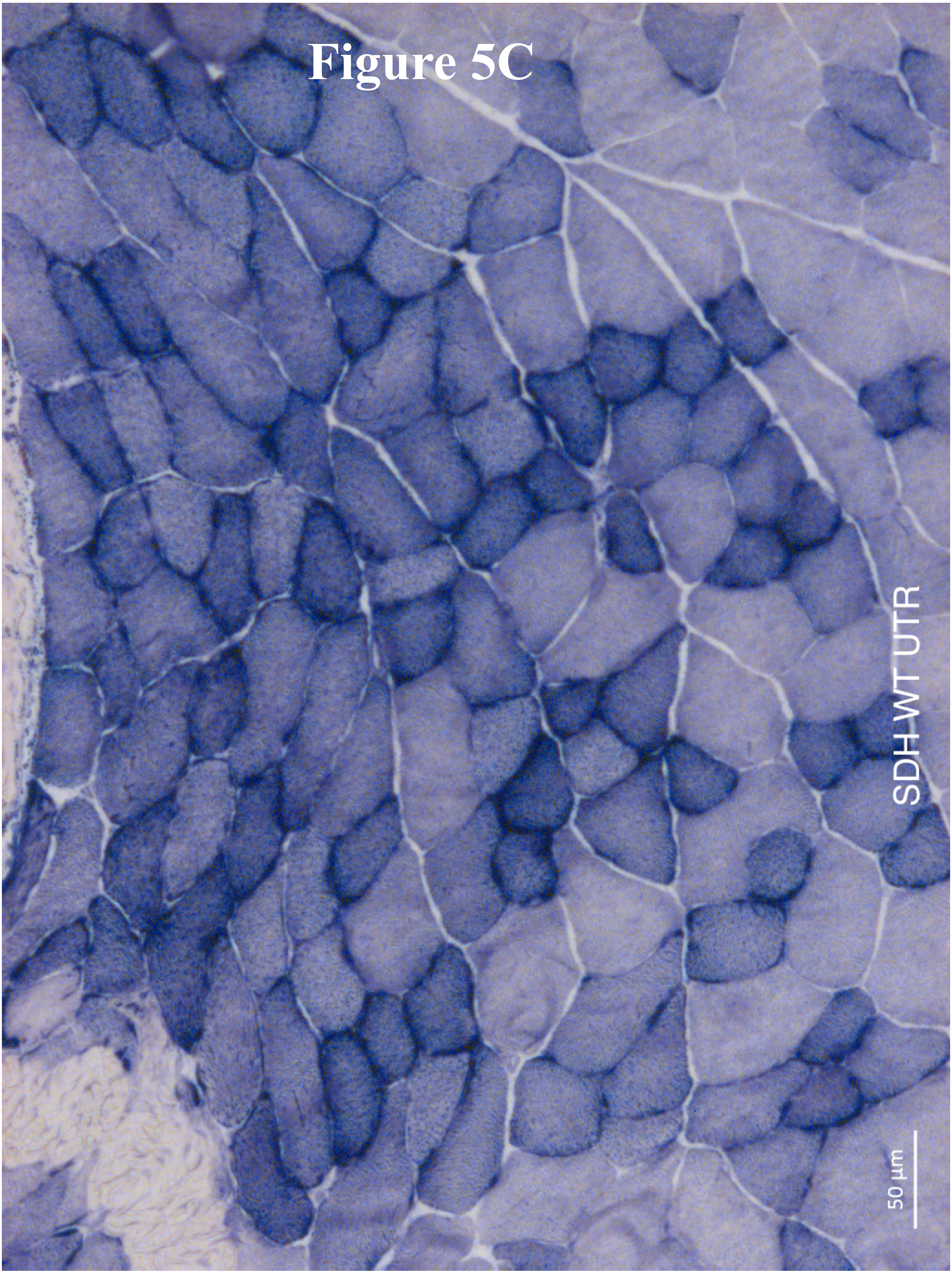

Supplement: Supplementary file 7 — Source Data for Figure 5 [file EMMM-10-e8799-s005.pdf]
